# Impact of distinct dystrophin gene mutations on behavioral phenotypes of Duchenne muscular dystrophy

**DOI:** 10.1242/dmm.050707

**Published:** 2024-12-24

**Authors:** Amel Saoudi, Manuela D. Mitsogiannis, Faouzi Zarrouki, Claire Fergus, Erwina Stojek, Silvia Talavera, Dervla Moore-Frederick, Vincent P. Kelly, Aurélie Goyenvalle, Federica Montanaro, Francesco Muntoni, Jack A. Prenderville, Ewa Sokolowska, Cyrille Vaillend

**Affiliations:** ^1^ CNRS, Institut des Neurosciences Paris-Saclay, Université Paris-Saclay, 91400 Saclay, France; ^2^ UVSQ, Inserm, END-ICAP, Université Paris-Saclay, Versailles, France; ^3^ Transpharmation Ireland Ltd, Trinity College Institute of Neuroscience, Trinity College Dublin, Dublin 2, Dublin, Ireland; ^4^ School of Biochemistry and Immunology, Trinity Biomedical Sciences Institute, Trinity College Dublin, Dublin 2, Dublin, Ireland; ^5^ Great Ormond Street Institute of Child Health, Dubowitz Neuromuscular Centre, University College London, London, United Kingdom; ^6^ Transpharmation Poland Sp. z o.o., Faculty of Veterinary Medicine, University of Warmia & Mazury in Olsztyn, 00-131 Olsztyn, Poland

**Keywords:** Brain dystrophin, *Mdx* ^5cv^, *Mdx52*, Dp140, Cognition, Emotional reactivity

## Abstract

The severity of brain comorbidities in Duchenne muscular dystrophy (DMD) depends on the mutation position within the *DMD* gene and differential loss of distinct brain dystrophin isoforms (i.e. Dp427, Dp140, Dp71). Comparative studies of DMD mouse models with different mutation profiles may help to understand this genotype−phenotype relationship. The aim of this study was (1) to compare the phenotypes due to Dp427 loss in *mdx^5cv^* mice to those of *mdx52* mice, which concomitantly lack Dp427 and Dp140; and (2) to evaluate replicability of phenotypes in separate laboratories. We show that *mdx^5cv^* mice displayed impaired fear conditioning and robust anxiety-related responses, the severity of which was higher in *mdx52* mice. Depression-related phenotypes presented variably in these models and were difficult to replicate between laboratories. Recognition memory was unaltered or minimally affected in *mdx^5cv^* and *mdx52* mice, at variance with the cognitive deficits described in the original Dp427-deficient *mdx* mouse, suggesting a difference related to its distinct genetic background. Our results confirm that Dp140 loss may increase the severity of emotional disturbances, and provide insights on the limits of the reproducibility of behavioral studies in DMD mouse models.

## Teaser

**Summary:** This study reports the behavioral phenotypes of two Duchenne muscular dystrophy mouse models lacking one or several dystrophin isoforms, highlighting the main outcome measures and their reproducibility in distinct laboratories.

## INTRODUCTION

Duchenne muscular dystrophy (DMD) is an X-linked, recessive neuromuscular syndrome that affects 1:5000 male births (Mah et al., 2014). DMD is caused by mutations in the *DMD* gene that comprises of 79 exons and presents seven internal independent promoters controlling the expression of distinct dystrophin protein isoforms (Dps) (Hoffman et al., 1987). Dps differ in size but share a C-terminal region containing binding sites for β-dystroglycan, dystrobrevin and syntrophin. Their tissue- and/or cell type-dependent expression patterns suggest they play different roles in different spatiotemporal contexts. Nevertheless, all of them participate in the clustering of different cell-membrane receptors and ion channels at the cell membrane (Perronnet and Vaillend, 2010). Full-length dystrophin isoform (Dp427) is expressed in both skeletal muscle fibers and in brain inhibitory synapses (Chelly et al., 1990; Górecki et al., 1992; Byers et al., 1993; Knuesel et al., 1999). Shorter dystrophins are also expressed in the nervous system, namely Dp260 (retina), Dp140 (brain), Dp116 (Schwann cells) and Dp71 (brain and retinal astrocyte endfeet) (Byers et al., 1993; Lidov et al., 1995; Wersinger et al., 2011; Barboni et al., 2023).

DMD is primarily characterized by progressive skeletal muscle weakness and wasting due to Dp427 loss in muscles (Mercuri et al., 2019). Central nervous system (CNS) comorbidities represent another important aspect of the DMD pathology. However, the relationship between specific brain dysfunctions and loss of one or more central dystrophins has yet to be fully elucidated owing to the complexity of the brain phenotype(s) and multiple dystrophins expressed in this tissue (Ricotti et al., 2016; Colombo et al., 2017). Clinical studies report impaired cognitive function with reduced learning and memory performances as well as attention deficits. IQ scores of patients with DMD are lower compared to those of the general population, and reach disability levels (IQ <70) in about one third of individuals (Billard et al., 1992; Cotton et al., 2001; Hinton et al., 2001, 2004; Taylor et al., 2010). Moreover, DMD is associated with several internalizing problems including stress reactivity, anxiety and depression as well as externalizing problems, such as emotional dysregulation and aggressiveness (Ricotti et al., 2016; Colombo et al., 2017). The CNS defects are heterogeneously expressed across the patient population in terms of type and the severity of each phenotype. Importantly, proximal mutations that prevent the expression of Dp427 alone are associated with milder neurological alterations, whereas more distally located mutations resulting in loss of additional brain dystrophins correlate with more-severe cognitive and behavioral comorbidities (Desguerre et al., 2009; Taylor et al., 2010; Maresh et al., 2023; Ricotti et al., 2016).

Functional studies of different mouse models of DMD may advance our understanding of how CNS functions are affected according to the position of the mutation within the *DMD* gene and, consequently, the loss of one or multiple dystrophins. The most widely studied DMD model is the *mdx* mouse, which carries a proximal non-sense mutation (exon 23) that prevents expression of Dp427 (Sicinski et al., 1989). At young-adult stage, this mutant mouse displays mild motor dysfunctions compared to those in humans diagnosed with DMD, due to effective regeneration processes compensating muscle fiber necrosis (Pastoret and Sebille, 1995). However, it shows emotional disturbances, including a marked enhancement of stress reactivity, and a subtle profile of hyperstimulation anxiety and mild cognitive impairments (Vaillend et al., 1995, 2004; Vaillend and Chaussenot, 2017; Engelbeen et al., 2021). Dp427 loss in this model specifically alters the GABAergic inhibitory system and disturbs the function of the fear circuitry, as *mdx* mice are characterized by abnormally high fearfulness in response to mild stress, and impairments in fear learning and memory (Sekiguchi et al., 2009; Vaillend and Chaussenot, 2017). Although comparative studies of DMD mouse models that lack other dystrophin isoforms due to more-distal mutations are still scarce, they are important to better understand genotype−phenotype relationships (Vaillend et al., 1998; Vaillend and Ungerer, 1999; Saoudi et al., 2021). We have previously compared the original *mdx* mouse model to the *mdx52* model that comprises a more distal *Dmd* mutation (Saoudi et al., 2021). In the latter, an out-of-frame deletion of exon 52 prevents expression of Dp260 in the retina, and of Dp140 − in addition to Dp427 − in brain. The *mdx52* model is of great relevance as it mimics genetic alterations found in 63% of patients with DMD (Béroud et al., 2007). We have reported more severely impaired emotional behaviors in *mdx52* compared to *mdx* mice (Saoudi et al., 2021). However, as *mdx* and *mdx52* mouse models are not generated by using the same genetic background, this could affect the expression of the phenotype.

In our present study, we aimed to further characterize genotype−phenotype relationships influencing brain-related comorbidities in DMD. We therefore compared the behavioral phenotype of the *mdx52* mouse with that of the *mdx^5cv^* mouse (Cox et al., 1993; Im et al., 1996). The latter was generated using the same genetic background as that used for the *mdx52* mouse model, and carries a proximal *Dmd* mutation (exon 10) that − similar to the original *mdx* model − only prevents expression of Dp427*.* Many laboratories may need to replicate the behavioral phenotypes of DMD mouse models in future preclinical studies. Indeed, several gene-therapy drugs have been developed and hold promise in treating the muscle pathology in DMD. One next challenge is to determine whether these drugs could have beneficial effects on certain cognitive and behavioral deficits associated with the disease. To evaluate the ease of replication, our investigation was carried out independently in two laboratories within the framework of a collaborative European project, at the Paris-Saclay Institute of Neuroscience, France (hereafter referred to as NeuroPSI) and Transpharmation Ireland Ltd, Ireland (hereafter referred to as TIL). We first found a markedly altered emotional behavior in mice of the *mdx52* model as compared to those of the *mdx^5cv^* model. We then addressed cognitive functions by using spatial and non-spatial recognition memory tests; in this case, no gross deficits were found in either mutant strains. Some of the emotional and cognitive deficits presented variably as compared to previous findings in the original *mdx* model, suggesting a putative influence of the genetic background. Thus, this present study sheds new light on the genotype−phenotype relationship in the pathogenesis of DMD, but also provides insights on the replicability of behavioral studies in rodent models of DMD.

## RESULTS

### Emotional reactivity

We have previously characterized an emotional hyper-reactivity phenotype more pronounced in *mdx52* mice (i.e. mice lacking Dp427 and Dp140) than in *mdx* mice (i.e. mice only lacking Dp427) (Saoudi et al., 2021). However, these two models had been bred on distinct genetic backgrounds, which might constitute a confounding factor (Beastrom et al., 2011). In our present study, we characterized the *mdx^5cv^* DMD mouse model in parallel with the *mdx52* model to better discriminate between the impact of the *Dmd* mutation profile and that of the genetic background on the expression of behavioral phenotypes. Indeed, the *mdx^5cv^* model carries a similar mutation profile to that of *mdx* but, like *mdx52* mice, *mdx^5cv^* mice are bred on a C57BL/6 instead a C57BL/10 genetic background (Im et al., 1996). Tests were performed in two laboratories (NeuroPSI, France; TIL, Ireland) to assess reproducibility and/or use of complementary equipment.

**Fig. 1.**
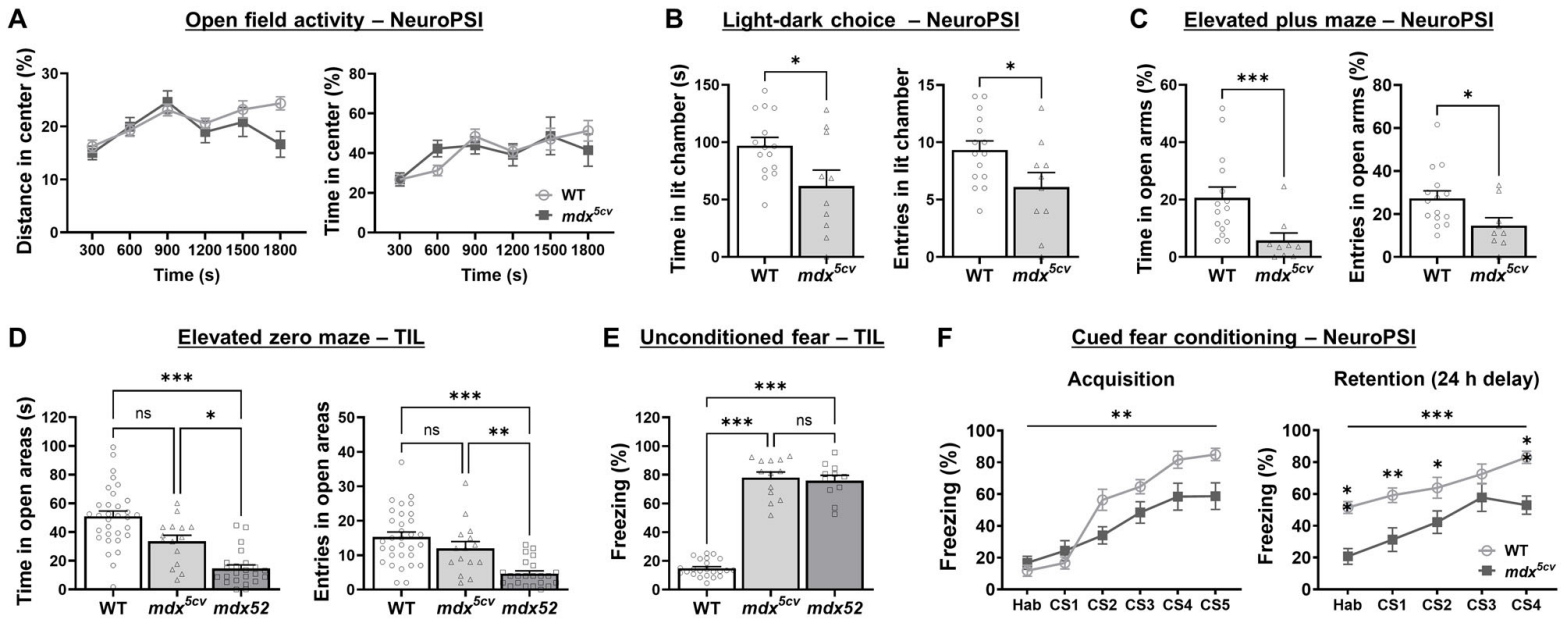
**Emotional reactivity and fear conditioning in *mdx^5cv^* and *mdx52* mice.** (A-C) Cohorts of 2-month-old *mdx^5cv^* mice (*n*=14) and their wild-type (WT) littermates (*n*=17) were submitted to a series of anxiety tests. Open-field activity (A). Plotted is the distance moved (left) or total time spent (right) within the center zone of the arena, expressed as a percentage of total distance moved or total time per 300 s time bin. Light−dark-choice test (B). Bar graphs show time (in s) spent in (left) or number of entries to (right) the lit box. Elevated plus maze test (C). Bar graphs show time spent in (left) or number of entries made to (right) open maze arms (both in %), normalized to total number of maze arm entries performed. (D) Elevated zero-maze test. Independent groups of 1.5–2-month-old *mdx^5cv^* (*n*=15), *mdx52* (*n*=24) and WT (*n*=33) mice underwent the elevated zero maze test. Bar graphs show time spent in (left) or entries to (right) the elevated zero maze (both in s). Anxiety indexes: time spent in open maze areas and number of entries made in open maze areas. (E) Unconditioned fear response test. Groups of 1.5–2-month-old *mdx^5cv^* (*n*=13), *mdx52* (*n*=11) and WT (*n*=23) that had first been tested in the elevated zero maze (see D) underwent the unconditioned fear response assay. Bar graphs show the total time of tonic freezing, i.e. immobility (in %), in response to a brief (15 s) scruff-restraint during a 5-min period. For WT animals from the two cohorts, results shown in D and E were pooled following verification that animal performance was statistically comparable. (F) Auditory-cued fear conditioning. Fear learning (acquisition) and fear memory (retention) were assessed in 3–4-month-old *mdx^5cv^* mice (*n*=14) and their WT littermate mice (*n*=17) that had previously undergone a set of anxiety tests (see A-C). Bar graphs show the learning performance as total time spent freezing (in %) during presentation of the conditioning stimulus (CS) − i.e. a 30-s audible tone (80 dB for 30 s, followed by a footshock) – repeated five times (CS1−CS5) during acquisition and four times (CS 1–CS4) during retention, which was performed 24 h later in a new context. A 1-week habituation period (Hab), a daily handling of all groups for 2 min twice a day for a week prior to the testing period. Data are presented as the mean±s.e.m. (A,F) or +s.e.m. (B-E). Statistical analyses: Mann–Whitney *U* test (B,C); Kruskal–Wallis test followed by Dunn's multiple comparisons test (D,E); two-way RM ANOVA followed by Šídák multiple comparisons test (A,F). *P<0.05; **P<0.01; ***P<0.001; ns, not significant. NeuroPSI, experiments carried out at the Paris-Saclay Institute of Neuroscience; TIL, experiments carried out at Transpharmation Ireland Ltd.

Similar experimental conditions that previously detected anxiety-related responses in *mdx* and *mdx52* mice were employed to assess a cohort of 2-month-old *mdx^5cv^* mice and their WT littermates at NeuroPSI. In the open field, mouse activity was evaluated during 30 min of free exploration. In this test, expression of anxiety-like behaviors was associated with avoidance of the center of the arena. As shown in Fig. 1A, the percentage of total distance travelled and time spent in the center of the open field by WT littermate and *mdx^5cv^* mice were comparable (Table S1), suggesting that *mdx^5cv^* mice show no emotional disturbances due to loss of Dp427 in this test. The same groups of mice were also subjected to two specific anxiety paradigms, the light−dark choice and use of the elevated plus maze. In the light−dark choice, mice had the choice to stay in a secure dark compartment or to explore an anxiogenic (brightly lit) compartment for 5 min (see Fig. S2B for schematic representations). As shown in Fig. 1B, *mdx^5cv^* mice exhibited enhanced anxiety-like behaviors as they entered the lit box less often and spent significantly less time in it compared to WT littermates (Table S1). In the elevated plus maze, the threat represented by the elevated open arms provides the anxiogenic stimulus. The time spent (in %) and entries to open arms made (in %) were significantly reduced for *mdx^5cv^* compared with WT mice (Fig. 1C, Table S1), confirming the presence of an enhanced-anxiety phenotype in *mdx^5cv^* mice. These two tests showed that anxiety-related behaviors can be clearly measured in Dp427-deficient *mdx^5cv^* mice. Interestingly, however, the anxiety phenotype appeared to be more robust in our present work than in a previous study using *mdx* mice, in which non-significant differences had been reported for several anxiety-related parameters (Saoudi et al., 2021).

In parallel, tests were performed at TIL, using the *mdx52* mouse model to determine the replicability of phenotypes previously observed at NeuroPSI (Saoudi et al., 2021). Groups of 3–4-month-old *mdx52* mice and their WT littermates were subjected to a 25-min open-field exploration under environmental conditions that were similar to those used at NeuroPSI (Fig. S2A, Table S1). Here, *mdx52* mice showed a similar total distance travelled or time spent (in %) in the center of the arena relative to WT mice; however, analysis within 5-min time bins demonstrated significantly reduced time (in %) spent in the center for *mdx52* versus WT littermates at ∼15 min after starting the assessment (Fig. S2A). This suggested presence of a transient or borderline hyper-anxious response in *mdx52* mice that was not observed in the *mdx^5cv^* model. Then, cohorts of 1.5–2 month-old *mdx52* mice and WT littermates were submitted to the light−dark choice by using a modified apparatus as compared to the one used at NeuroPSI (Fig. S2B). Despite environmental differences, *mdx52* mice tested at TIL also presented significantly increased indexes of anxiety (e.g. less time spent in the lit chamber) compared to those of WT littermates (Fig. S2C, left plot; Table S1). To assess the impact of age and retesting on this behavior, the same mouse groups were retested at 3–4 months of age, and were found to show the same behavioral abnormalities (Fig. S2C, right plot; Table S1). This suggests that age or repeated testing do not affect the phenotype observed when using this test.

To obtain a more direct comparison of emotional reactivity phenotypes across DMD mouse models, cohorts of 1.5–2 months-old *mdx^5cv^* and *mdx52* mice, and of their WT littermates were compared in same assays at TIL. When using the elevated zero-maze, an optimized version of the elevated plus maze, *mdx^5cv^* and *mdx52* mice spent less time in open areas and entered them less frequently compared to WT mice (Fig. 1D, Table S1). Notably, these behavioral measures were significantly reduced in *mdx52* compared to *mdx^5cv^* mice (Fig. 1D, Table S1). Overall, these results confirm a more marked emotional reactivity in *mdx52* mice. The effects of age and retesting were also assessed by resubmitting the animals to the test at 3–4 months of age (Fig. S2D,E, Table S1). At this age, occurrence of the anxious phenotype appeared to be similar between DMD models (Fig. S2D, Table S1). Pairwise comparisons between genotype groups at stages of young and mature adult revealed a decrease in time spent exploring the open areas for all genotypes, with significant post hoc differences for WT and *mdx^5cv^* animals (Fig. S2E, Table S1). Hence, increased age and/or repeated testing could play a role on the expression of anxiety-like behaviors when using the elevated zero maze.

The same groups of mice were then subjected to manual restraint to evaluate their unconditioned fear response. Fearful behavior was reflected by the time (in %) spent in tonic immobility (freezing) after a scruff-restraint period of 15 s during the 5-min testing period. Both mouse models showed comparable levels of freezing (∼80%) and demonstrated a significantly enhanced fear response compared to the WT group that only exhibited a short duration of immobility (<20%) (Fig. 1E, Table S1). Excessive freezing was also observed in *mdx^5cv^* and *mdx52* mice retested at 3–4 months of age (Fig. S2F, Table S1). Moreover, significantly increased levels of freezing were detected in pairwise comparisons between young and adult stages for both mutant groups but not for the WT group, indicative of a potentially exacerbating effect of age on this phenotype (Fig. S2G, Table S1).

### Cued fear conditioning

Emotional learning and memory were explored in 4-month-old *mdx^5cv^* mice at NeuroPSI in the context of an amygdala-dependent Pavlovian association paradigm (auditory-cued fear conditioning), since both *mdx* and *mdx52* mouse models have been reported to show learning and memory impairments in this task (Vaillend and Chaussenot, 2017; Saoudi et al., 2021). To test these functions in *mdx^5cv^* mice, we used the same protocol as employed in the aforementioned studies. All mice first underwent a single acquisition session comprising five trials, during which a 30-s audible tone, i.e. conditioning stimulus (CS), was paired with a foot-shock, i.e. unconditioned stimulus (US). Fear memory retention was measured 24 h later in a 4-trial CS-only session. Robust freezing upon exposure to the auditory tone (CS) normally reflects successful associative fear learning and memory. In WT mice, freezing durations during acquisition gradually increased across successive trials (from CS1 to CS5) to reach ∼80% of total CS presentation time. In *mdx^5cv^* mice, freezing durations also progressively increased during acquisition but, overall, remained significantly lower than those of WT mice, suggesting a delay in fear learning (Fig. 1F, Table S1). Surprisingly, freezing levels of *mdx^5cv^* mutants could not reach those of WT mice even on the last trial (freezing <60%). During the retention session, high levels of freezing (∼50−80%) − reflecting robust fear memory − were measured in WT mice, while *mdx^5cv^* mice displayed significantly lower levels of freezing (∼20−50%), indicating deficits in memory recall performance, which might be related to their lower performance at the end of the acquisition session. Notably, during the habituation phase preceding the retention trials, freezing responses were significantly reduced in *mdx^5cv^* versus WT animals, suggesting an additional impairment of contextual fear memory.

### Spatial recognition memory in a T-maze

**Fig. 2.**
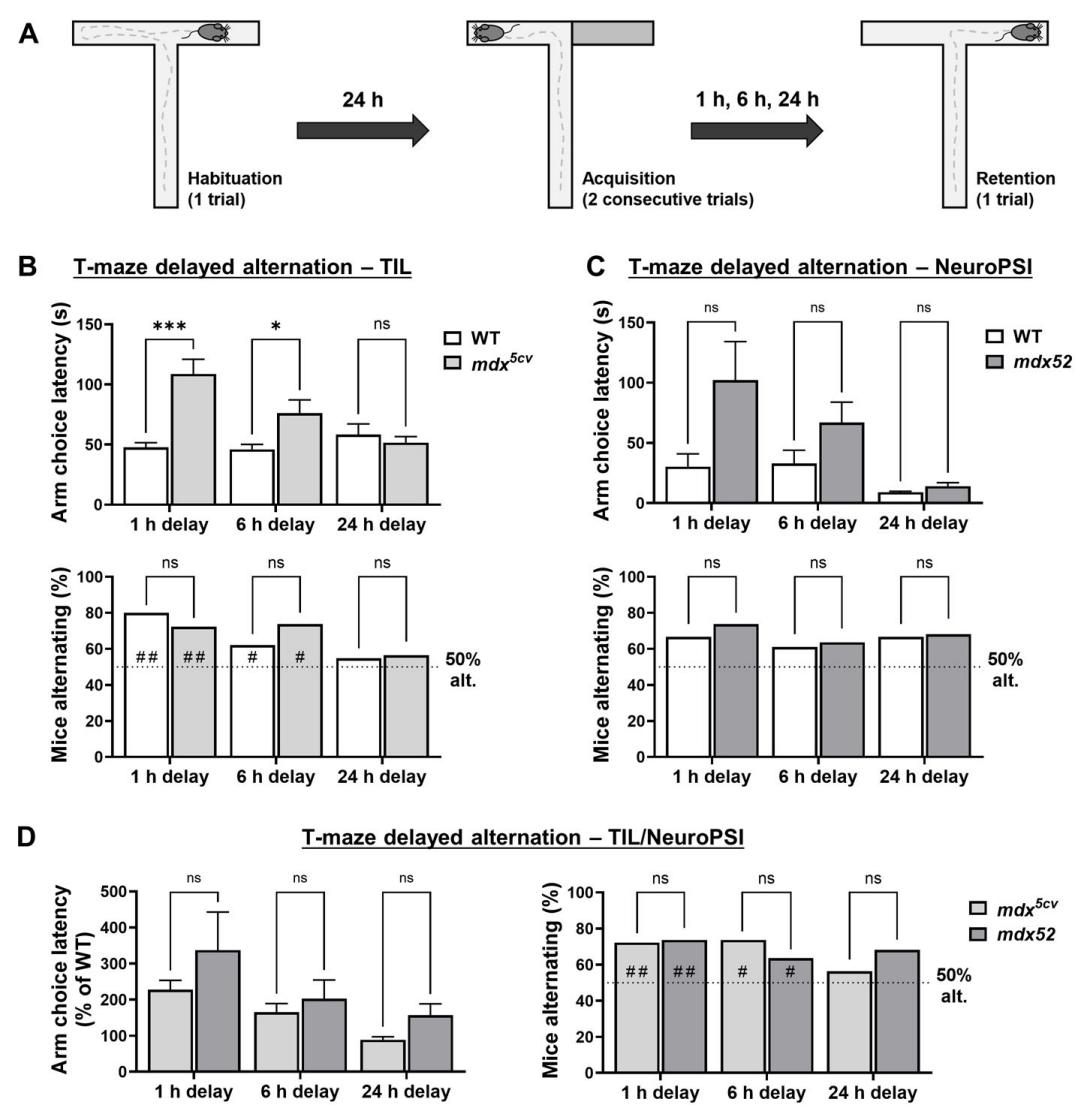
**T-maze spatial recognition memory in *mdx^5cv^* and *mdx52* mice.** (A) Schematic of the T-maze delayed alternation test. (B,C) Bar graphs showing the latency (in s) to choose a T-maze arm (top panels) or the number of mice alternating during the retention session (bottom panels), expressed as a percentage of total number of mice tested (chance level: 50%). Experiments were either performed at TIL (B) with 3–4-month-old *mdx^5cv^* mice (*n*=38) and wild-type (WT) littermates (*n*=31) or at NeuroPSI (C) with 3–4-month-old *mdx52* mice (*n*=22) and their WT littermates (*n*=23). (D) Bar graphs showing the latency of *mdx^5cv^* and *mdx52* mice to choose a T-maze arm as the percentage of the matched WT group (left) or the number of *mdx^5cv^* and *mdx52* mice (in %) alternating during the retention sessions in experiments performed at TIL and NeuroPSI. Latencies are expressed as the mean+s.e.m. and were analyzed using two-way RM ANOVA or the mixed-effects model (REML). Data are presented as the mean or +s.e.m. Significance values according to Šídák multiple-comparison post hoc tests: *P<0.05; ***P<0.001; ns, not significant. Chi-squared test was used to assess the percentage of mice alternating between genotypes and for comparison to chance level (50%). ^#^*P*<0.05; ^##^*P*<0.01; ns, not significant. Dotted horizontal lines indicate 50% of mice alternating (50% alt.). NeuroPSI, experiments carried out at the Paris-Saclay Institute of Neuroscience; TIL, experiments carried out at Transpharmation Ireland Ltd.

We further compared *mdx^5cv^* and *mdx52* mice in different cognitive tasks involving recognition memory, which is known to be impaired in the original *mdx* model (Vaillend et al., 1995, 2004; Sinadinos et al., 2015). In particular, *mdx* mice have previously been reported to exhibit impaired long-term spatial recognition memory (i.e. a 24 h delay) in a T-maze delayed alternation task (Vaillend et al., 1995). Here, this task was employed to compare *mdx^5cv^* and *mdx52* mouse models tested by using the same protocol at TIL and NeuroPSI, respectively. Mice were first submitted to a 2-trial acquisition session, in which they were forced to explore one of the two lateral arms of the T-maze. Memory retention was evaluated in a single trial using different post-acquisition delays (1, 6 or 24 h), by quantifying the percentage of mice successfully alternating their choice of T-maze arm between acquisition and retention (Fig. 2A).

In *mdx^5cv^* mice, latencies to choose a lateral arm during the retention session were significantly higher compared to those of WT littermates (Fig. 2B, top; Table S1). A similar phenotype was observed in *mdx52* animals; although, due to larger inter-individual variability, no significant post hoc differences were identified between mutant and WT regarding specific delays (Fig. 2C, top; Table S1). These data provide additional evidence of enhanced emotional reactivity characterizing both DMD models. Statistical analyses of choice latencies during acquisition sessions corroborated this hypothesis for both *mdx^5cv^* (Fig. S3A, Table S1) and *mdx52* mice (Fig. S3B, Table S1). It is possible that tail-handling stress during acquisition induced fear, anxiety and risk-aversion in test mice, consequently, affecting exploratory activity and resulting in longer choice latencies during retention, particularly in case of shorter post-acquisition delays.

Spontaneous alternation rates measured in *mdx^5cv^* mice confirmed that short-term spatial reference memory is not impaired in Dp427-deficient mice. Both *mdx^5cv^* mutant and WT animals displayed comparable − i.e. significantly above chance (50%) – levels of alternation rates during retention sessions performed after delays of 1 h and 6 h (Fig. 2B, bottom; Table S1). However, results at a 24 h delay did not allow replication of previous findings in the *mdx* model concerning long-term memory deficits, since both WT and *mdx^5cv^* mice demonstrated alternation rates that were similarly close to chance level, i.e. an absence of quantifiable spatial recognition memory. The *mdx52* animals and their WT littermates also showed comparable alternation rates at retention, which were slightly but not significantly above chance levels (∼60–70% of correctly alternating mice) at 1 h, 6 h and 24 h (Fig. 2C, bottom; Table S1). Moreover, statistical comparison of the two mouse models at all delays did not reveal any significant differences of either latency regarding choice of T-maze arm or alternation percentage (Fig. 2D). Despite non-significant results in pairwise statistical analyses performed for each individual delay, an effect of genotype was detected in a cross-delay comparison of latencies regarding choice of T-maze arm, with scores of *mdx52* mice being overall higher than those of *mdx^5cv^* mice (Table S1). These findings highlight an enhanced emotional response of the *mdx52* model compared to the *mdx^5cv^* mutant, which is in line with previous observations in the assays investigating fear and anxiety described above.

### Spatial and non-spatial object recognition memory

**Fig. 3.**
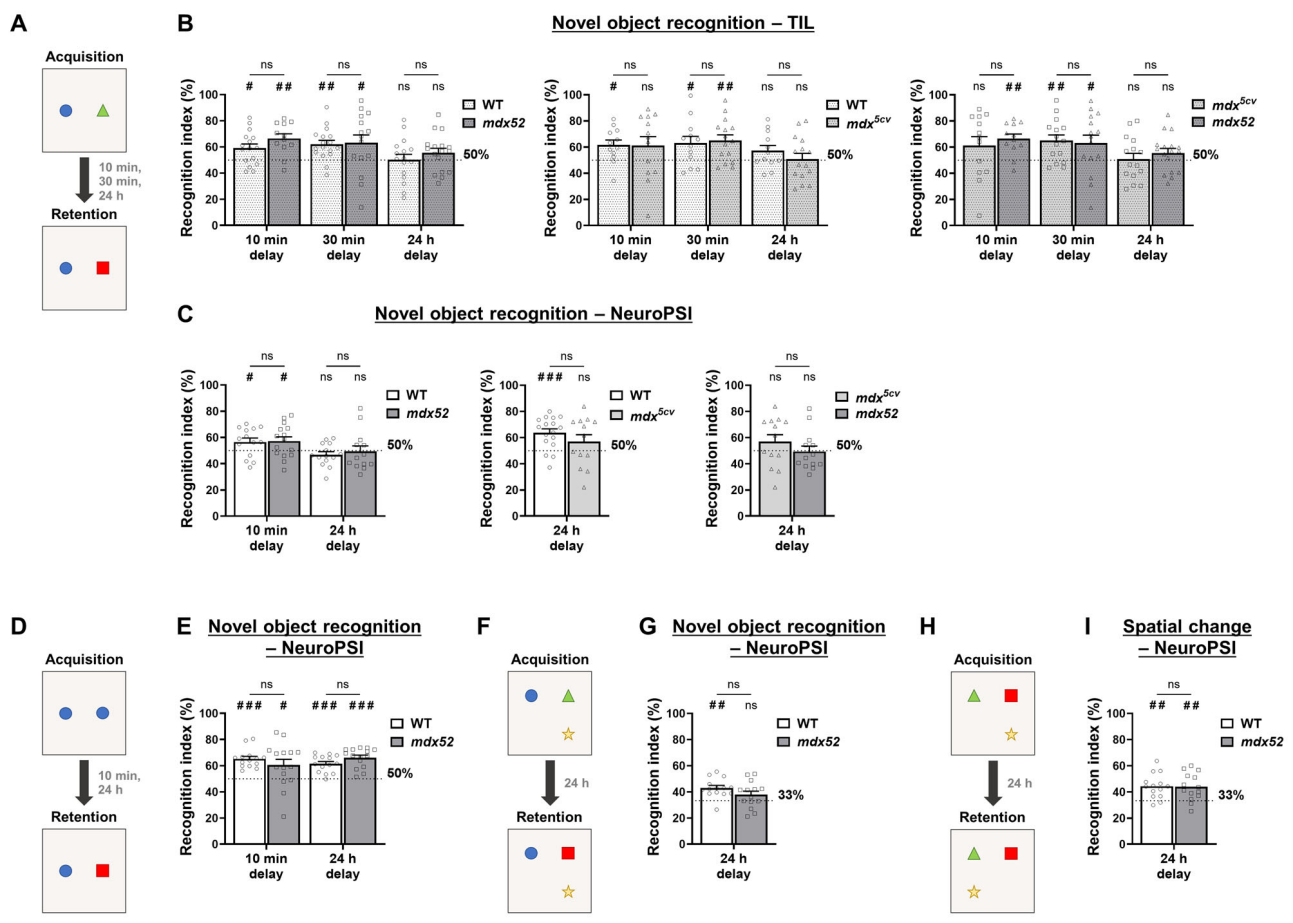
**Non-spatial and spatial object recognition memory in *mdx^5cv^* and *mdx52* mice.** (A-C) Novel-object-recognition test using two different objects during the acquisition period. Schematic of the protocol used (A). (B) Bar graphs showing the recognition index (RI) values, i.e. the recognition memory during retention, (in %) at 10 min, 30 min or 24 h delay as indicated. Left panel: RIs obtained from 3–4-month-old *mdx52* mice (*n*=18) and wild-type (WT) littermates (*n*=18). Middle panel: RIs obtained from 3–4-month-old *mdx^5cv^* mice (*n*= 14) and their WT littermates (*n*=14). Right panel: comparison of the results obtained from the two mutant mouse groups shown in left and middle panels. All tests were performed at TIL. (C) Novel object recognition test performed at NeuroPSI by using the same protocol as described in A at 10 min or 24 h delay as indicated. Left panel: RIs of 3–4-month-old *mdx52* mice (*n*=16) compared to those of their WT littermates (*n*=14). Middle panel: RIs of 3–4-month-old *mdx^5cv^* mice (*n*= 14) with their WT littermates (*n*=17). Right panel: comparison of the results obtained from the two mutant mouse groups shown in left and middle panels at 24 h delay (right plot). (D,E) Novel object recognition test with two identical objects set at acquisition. Schematic of the protocol used (D). (E) RIs at 10 min and 24 h delay of 3–4-month-old *mdx52* mice (*n*=16) and their WT littermates (*n*=14). (F-I) Novel object recognition (F,G) and spatial object recognition (Spatial change) tests (H,I) by using three different objects set at acquisition at 24 h delay. Schematic of the protocols used are shown in F and H. Either one object was replaced with a new one at retention (F,G) or one object was moved at retention (H,I). Bar graphs show the respective RIs of 3–4-month-old *mdx52* mice (*n*=14) and their WT littermates (*n*=14) (G,I). Data are presented as the mean+s.e.m. RIs were calculated as the time spent exploring the novel or moved object over the total object-exploration time measured during the 5 min retention trial (in %). Statistical analyses: two-way RM ANOVA or mixed-effects model (REML) followed by Šídák multiple comparisons test (B,C left plot, E) or Mann–Whitney *U* test (C,G,I) for simple genotype comparisons (all not significant). Wilcoxon signed-rank test was used for comparison to chance level [with 50% in two-object test (E) and 33.33% in three-object tests (G,I): ^#^*P*<0.05; ^##^*P*<0.01; ^###^*P*<0.001; ns, not significant]. NeuroPSI, experiments carried out at the Paris-Saclay Institute of Neuroscience; TIL, experiments carried out at Transpharmation Ireland Ltd.

Previous research has also established the presence of long-term (24 h) deficits of object recognition memory in the *mdx* mouse model (Vaillend et al., 2004). Therefore, both at TIL and NeuroPSI, we subjected groups of 3–4-month-old *mdx52* and *mdx^5cv^* mice, together with their respective WT littermates, to multiple novel object recognition tests performed at different post-acquisition delays (10 min, 30 min, 24 h), using a protocol similar to that previously employed for *mdx* mice (Fig. 3). First, mice were exposed to two different objects during an acquisition session, then one object was replaced with a new one during the retention session (Fig. 3A). Recognition memory during retention was evaluated by using the recognition index (RI), with robust memory recollection being reflected by RI values significantly above 50% (chance level).

At TIL, RIs measured for both *mdx52* and *mdx^5cv^* mouse groups were significantly higher than chance level at 10 min and 30 min delays, without any significant effect of the genotype (Fig. 3B, left and middle panels; Table S1), confirming that short-term recognition memory is preserved in these DMD mouse models. At 24 h delay, no significant difference from chance level was detected for either genotype, indicating an overall absence of long-term object recognition memory that is independent of the *Dmd* mutation profile (Fig. 3B, left and middle panels; Table S1). Memory performance of the two DMD models did not differ significantly at any delay (Fig. 3B, right panel). This was confirmed in *mdx52* mice by experiments similar to those conducted at NeuroPSI (Fig. 3C, Table S1). For *mdx^5cv^* mice, tests were only performed with a 24 h retention delay at NeuroPSI. The WT group performed above chance level, while performance of the *mdx^5cv^* group did not differ from chance level. However, there was no main genotype effect. *Mdx^5cv^* mice, thus, showed a trend for impaired recognition memory that resembles the deficit displayed by the original *mdx* mouse; but, the inter-individual variability did not allow consistent and robust reproduction of such findings when using this assay. Performances of *mdx^5cv^* and *mdx52* mice at the 24 h retention delay did not differ from chance level, and were statistically comparable (Fig. 3C, right panel).

Overall, compared with those previously reported in the original *mdx* mouse (Vaillend et al., 2004; Zarrouki et al., 2022), these results suggest this protocol not being optimal for mice on a C57BL/6 genetic background. We, thus, attempted to simplify the protocol by using a set of two identical objects during acquisition (Fig. 3D). Indeed, under these conditions, the RIs quantified for both *mdx52* and WT groups were significantly different from chance level at both 10 min and 24 h delays (Fig. 3E, Table S1). However, no significant RI differences were detected between *mdx52* and WT littermates, reflecting a failure to replicate the impaired long-term memory that had been reported in *mdx* mice when following this new protocol (Vaillend et al., 2004; Zarrouki et al., 2022).

In the novel object recognition protocols described above, which included a 1-week habituation period to the test arena, we observed that the mice were frequently climbing on objects and jumping from objects or against the walls of the arena. Consequently, we hypothesized a low interest for the task that might have influenced the results above. NeuroPSI further tried to optimize the assay by shortening the habituation phase to a single day. A naive group of *mdx52* mice and their WT littermates was then submitted to an alternative protocol, involving exposure to a set of three different objects during acquisition and replacement of one object with a new one during retention (Fig. 3F). The single-trial acquisition session was divided into three consecutive short trials separated by brief resting intervals. Object recognition memory was assessed 24 h later. In this protocol, the RIs measured for WT mice − but not *mdx52* mice − were significantly higher than 33% (i.e. the chance level when using three objects) (Fig. 3G, Table S1). Despite this, no significant RI differences were detected between the *mdx52* and WT groups, indicating only a subtle impairment in long-term recognition memory in this DMD mouse model.

The same refined three-object protocol was then employed to examine spatial object recognition memory in *mdx52* mice. In this test, one of the three objects used for acquisition was moved rather than replaced during the retention session (Fig. 3H). The RIs quantified in both genotype groups were comparably higher than chance level (33%) (Fig. 3I, Table S1). In line with the results of the alternation task above, this experiment did not reveal any spatial object-recognition memory deficits in the *mdx52* mouse model.

Besides memory performance, we found that *mdx52* mice explored objects significantly less than WT littermates during acquisition sessions of tests performed with two objects (Fig. S4A-D, Table S1). In contrast, in tests performed using three objects, exploration times were comparable between genotypes (Fig. S4E-H, Table S1), which supports the hypothesis that the original protocol used for *mdx* mice had to be adjusted for memory testing in *mdx52* mice. Yet, these changes did allow detecting significant memory impairments.

### Helplessness and depressive-like behaviors

Depressive-like behaviors were investigated by using standard evaluation of behavioral despair and learned helplessness in tail suspension and forced swimming tests, in which depression-like states are typically reflected by an increase and earlier onset of immobility (freezing) episodes.

**Fig. 4.**
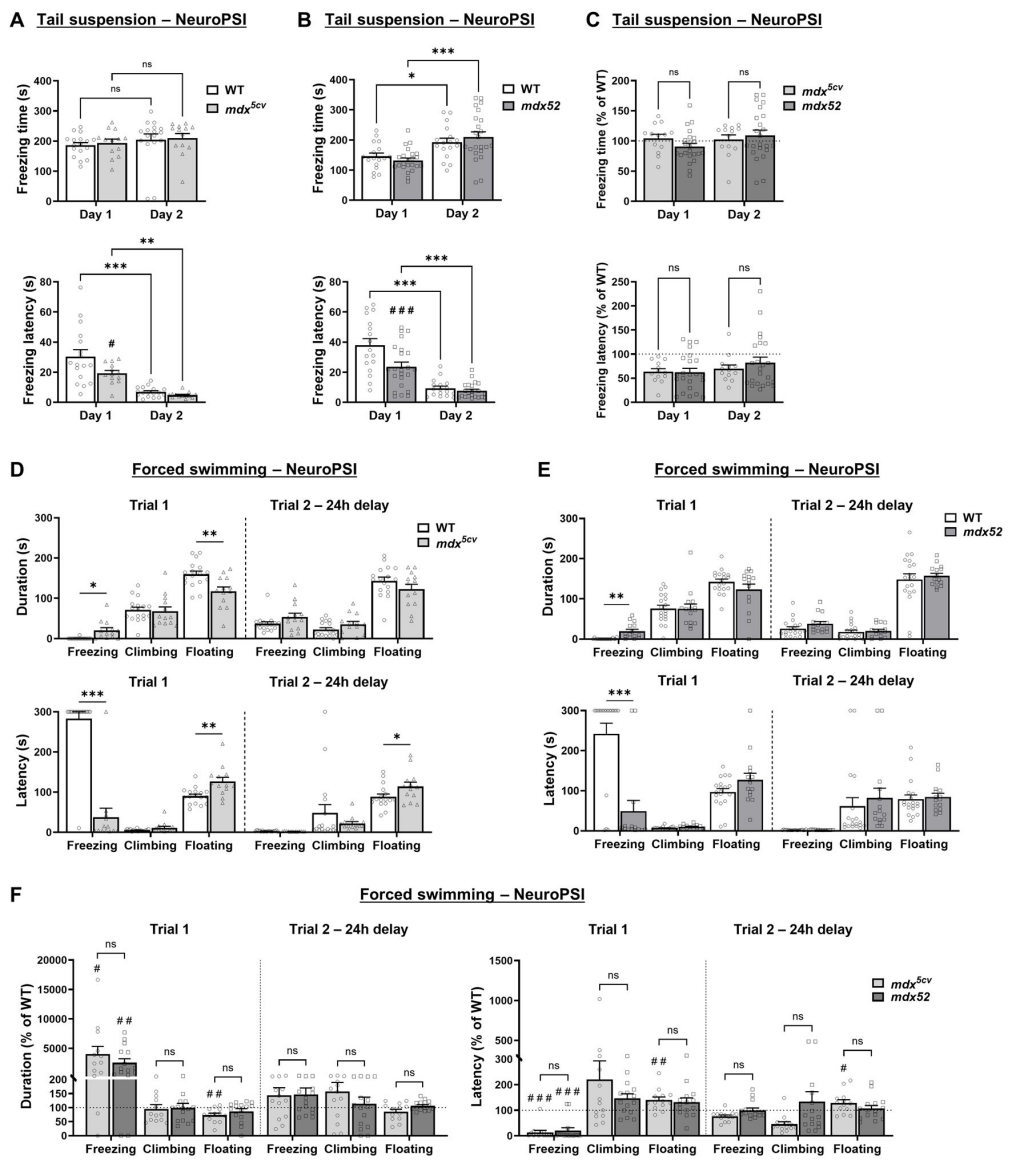
**Behavioral despair and learned helplessness in *mdx^5cv^* and *mdx52* mice.** (A-C) Tail suspension tests performed at NeuroPSI with groups of 3–4-month-old *mdx^5cv^* mice (*n*=13) and their wild-type (WT) littermates (*n*=17) (A), and groups of *mdx52* mice (*n*=23) and their WT littermates (*n*=17) of corresponding age (B). Duration of freezing, i.e. immobility, and latency were measured during two consecutive days of testing (Day 1, Day 2). For each parameter, comparisons between the two DMD mouse models are shown in C. (D-F) Forced swimming test of 3–4-month-old *mdx^5cv^* (*n*=13) and their WT littermates (*n*=17) (D), and in 3–4-month-old *mdx52* mice (*n*=15) and their WT littermates (*n*=19) (E) carried out at NeuroPSI. Duration of freezing, climbing and floating, and of latency (in seconds) were measured in two successive trials that had been 24 h apart (Trial 1, Trial 2 −24h delay). (F) For each DMD mouse model, duration and latency regarding each parameter are plotted in comparison to their WT littermates (% of WT). Data are presented as the standard error of the means (+s.e.m.), using two-way repeated measures ANOVA. Significance (Šídák multiple-comparison post hoc test): *P<0.05; **P<0.01; ***P<0.001; ns, not significant. Comparisons to WT littermate levels: ^#^*P*<0.05; ^##^*P*<0.01; ^###^*P*<0.001. NeuroPSI, experiments carried out at the Paris-Saclay Institute of Neuroscience; TIL, experiments carried out at Transpharmation Ireland Ltd.

Groups of 3–4-month-old *mdx^5cv^* and *mdx52* mice, and their corresponding WT littermates, were subjected to both tests at NeuroPSI. Assays consisted of two trials separated by a 24 h interval. In the tail suspension test, time spent freezing was comparable between *mdx^5cv^* and WT mice in the two test trials (Fig. 4A, top; Table S1). However, *mdx^5cv^* mice displayed shorter latencies of freezing compared to WT mice on the first trial (day 1) (Fig. 4A, bottom; Table S1), suggesting enhanced behavioral despair in this model lacking Dp427. On the second trial (day 2), both genotypes exhibited shorter freezing latencies relative to the first trial, indicating unaltered learned helplessness in *mdx^5cv^* mice. The *mdx52* animals also showed comparable freezing durations relative to WT littermates and comparable learned helplessness (day 1 versus day 2), but a reduction in freezing latencies compared to WT littermates on the first day of testing, corroborating findings of an association between Dp427 and higher expression of behavioral despair. Comparing performance of the two models did not reveal statistical differences, which confirms that the additional loss of Dp140 in *mdx52* mice did not further increase behavioral despair (Fig. 4C).

In the forced swimming test, *mdx^5cv^* mice exhibited reduced freezing latencies and extended freezing durations compared to those of WT littermates on the first trial, an additional indication of enhanced behavioral despair (Fig. 4D, Table S1). However, *mdx^5cv^* mutants also showed extended floating latencies and reduced floating durations versus WT mice. Learned helplessness as reflected by reduced latencies to freezing and increased freezing times was observed for both genotypes on the second trial. Reduced latency to freezing and increased freezing duration in a first trial of forced swimming were also observed in the *mdx52* model (Fig. 4E, Table S1), confirming presence of enhanced behavioral despair. Again, learned helplessness assessed 24 h later was unimpaired in *mdx52* mice. Finally, when comparing performance of the two DMD models we did not find statistical differences, confirming that *mdx^5cv^* and *mdx52* mice displayed comparable phenotype in this test (Fig. 4F).

Interestingly, a cohort of 3–4-month-old *mdx52* and WT littermates was also investigated at TIL by using this test. In contrast with the mice tested at NeuroPSI, the *mdx52* group did not present reduced latency and increased duration of freezing in the first trial (Fig. S5, Table S1). Considering the discrepancies in the findings between our two laboratories − which may, in part, depend on environmental conditions and specific experimental factors − we could not demonstrate easy reproducibility of these phenotypes.

## DISCUSSION

In this multicenter comparative study, we first confirmed the genotype−phenotype relationship between the location of *Dmd* mutations and the extent of emotional alterations in DMD mouse models. Comparing *mdx52* and *mdx^5cv^* mouse models, which share a common genetic background, was successful in isolating the contribution the additional loss of Dp140 has on emotional deficits. Indeed, we found that *mdx52* mice, which lack both Dp427 and Dp140, display a more severe and robust anxiety phenotype than *mdx^5cv^* mice, which only lack Dp427. These findings are in line with recent literature on patients with DMD (Maresh et al., 2023) and, overall, suggest that anxiety-related phenotypes are more negatively affected by distal mutations that prevent expression of multiple brain dystrophins.

We also addressed the inter-lab replicability of anxiety- and fear-related phenotypes in DMD mouse models, and the possibility to observe these phenotypes at different ages in test−retest experiments. This is relevant for future preclinical studies, in which testing before and after treatment administration may introduce confounding factors in the interpretation of experimental data. In our two laboratories, we showed that open-field testing may only reveal borderline behavioral changes, while tests in which anxiety is triggered by a brightly lit environment (light−dark choice) or void avoidance (elevated plus maze and zero maze) enable reliable quantification of anxiety-related disturbances. Importantly, these deficits showed easy reproducibility even when slightly different equipment and protocols were used at the two locations. We also showed that phenotypes displayed by young (2-month-old) DMD mouse models in light−dark choice and zero maze tests can still be observed when mice are retested at 3−4 months of age. However, it is noteworthy that the more severe phenotype detected in *mdx52* mice when using the zero maze was only observable during the initial test carried out with young mice. Regarding the most robust phenotype reported in dystrophic mouse models – i.e. the restraint-induced unconditioned fear response − Sekiguchi and colleagues showed its progressive increase during the postnatal period in *mdx* mice, reaching a plateau (80−90% of freezing) at ∼3 months of age (Sekiguchi et al., 2009). Here, we show that the amount of post-restraint freezing significantly increases between 2 and 3−4 months of age in both *mdx^5cv^* and *mdx52* mice. This phenotype appears to be common to both mutants, with comparable magnitude at both ages.

Depressive-like behaviors are part of the internalizing problems displayed by some patients with DMD (Ricotti et al., 2016; Colombo et al., 2017). Previous work in *mdx* mice did not reveal obvious depression-related behavior, and significant changes in some parameters of the forced swimming test (capacity to stay afloat, escape-oriented climbing) rather suggested a main influence of stress and/or motor impairments on performance (Vaillend and Chaussenot, 2017). Here, we found evidence of increased behavioral despair in both *mdx^5cv^* and *mdx52* mice. In the tail suspension and forced swimming tests, these two models comparably displayed shorter latencies and longer durations of freezing episodes compared to WT, suggesting that Dp140 does not additionally affect these behaviors. However, depressive-like phenotypes in forced swimming test were not consistently observed in our two laboratories, a finding that might be related to subtle discrepancies between protocols (test room temperature, size of cylinder) or to differences in basal stress levels in the two animal facilities (depending on housing and/or handling conditions). It might be worth considering the possible influence of animal husbandry conditions that differed between our two laboratories: for instance, mice were kept in individually ventilated cages (IVCs) throughout the study at TIL, while they were transferred to standard cages after weaning at NeuroPSI. Indeed, several studies have pointed to the influence of housing conditions on spontaneous and disease-relevant mouse behaviors (Logge et al., 2014; Pasquarelli et al., 2017; Polissidis et al., 2017).

Impaired Pavlovian fear learning and memory, as assessed in the auditory-cued fear-conditioning paradigm, is another robust phenotype, and we showed here that it is readily observable in *mdx^5cv^* mice as it is in other DMD mouse models; thus, confirming that it primarily depends on Dp427 loss. Addressing other types of memory, such as recognition memory, unexpectedly proved to be the most challenging task of this study. Indeed, neither of our two laboratories could fully replicate the impaired long-term recognition memory that has previously been reported in the original *mdx* mouse model (Vaillend et al., 1995, 2004; Sinadinos et al., 2015). In our current study, neither *mdx^5cv^* nor *mdx52* mice were found to have spatial memory deficits in the delayed alternation task when using a T-maze at different retention delays. In the object recognition test, *mdx^5cv^* mice showed a trend towards *mdx* mice-like impairments in long-term (24 h) recognition memory. However, the inter-individual performance variability between *mdx^5cv^* and WT littermates did not allow detection of a significant genotype effect in this test. Similarly, *mdx52* mice displayed subtle long-term recognition memory deficits also associated with high inter-individual variability. Overall, these findings suggest that, in order to consistently identify an effect of *Dmd* mutations on recognition memory, very large samples would be required in these assays. We further tested whether a shortening of the habituation phase would improve the protocol, as this facilitated longer periods of object exploration during the acquisition and retention phases for mutant mice. However, we again observed a trend towards long-term recognition memory deficits in both *mdx^5cv^* and *mdx52* mice compared to WT littermates, but no significant genotype effect. In addition, others have already reported difficulties in phenotype replication among partners' laboratories, despite the use of similar protocols (Hayward et al., 2022). In summary, recognition memory performance is likely to be affected by Dp427 deficiency but − due to inter-individual variability rendering the use of large groups of animals necessary − cannot be considered as an optimal and robust behavioral outcome to adopt in preclinical studies.

Interestingly, our study provides converging results suggesting that the genetic background of *Dmd* mouse models may substantially modulate expression of DMD-associated phenotypes. This is based on the comparison of the present results with our previous reports on the original Dp427-deficient *mdx* mouse. However, we used identical equipment, protocols and experimenters in these distinct studies, which supports the relevance of our observations. The *mdx* and *mdx^5cv^* models were, respectively, generated on a C57BL/10 and C57BL/6 background but, since both models solely lack the Dp427 isoform, their behavioral profiles were expected to be similar. However, our present study, using *mdx^5cv^* mice, did not lead to a complete replication of the phenotypes observed in the original *mdx* mouse. From an emotional reactivity perspective, *mdx^5cv^* mice demonstrated moderate hyper-anxiety but, clearly, showed more robust impairment than *mdx* mice. This suggests that anxiety phenotypes are more markedly and consistently expressed on a C57BL/6 genetic background in DMD mouse models, and was confirmed in our two laboratories, despite variations in experimental conditions and setups. Some of our present data in depression-related tests suggested enhanced expression of behavioral despair in *mdx^5cv^* mice, which also differs with results previously obtained by using *mdx* mice (Vaillend and Chaussenot, 2017). Moreover, we have previously reported that, although cued fear learning in *mdx* mice is delayed, ultimately, it reaches WT levels by the end of the acquisition phase (Chaussenot et al., 2015; Vaillend and Chaussenot, 2017). In contrast, *mdx52* mice present slower progression of performance and fail to reach WT level during acquisition (Saoudi et al., 2021), suggesting that the additional loss of Dp140 in *mdx52* mice increases the severity of fear conditioning impairments. However, in our present study, *mdx^5cv^* mice lacking only Dp427 exhibited delayed learning curves that were comparable to those of *mdx52* mice, rather suggesting that fear conditioning impairments are more severe when mutations are expressed on a C57BL/6 genetic background. We also had difficulties confirming the recognition memory deficits in *mdx^5cv^* mice, which had originally been observed in *mdx* mice tested for object recognition and alternation behavior in a T maze, even after optimization of the protocol for the object recognition test.

Our results, suggesting that the genetic background may influence behavioral performance in DMD mouse models, are in line with many studies that point to differences between distinct C57BL strains in a variety of experimental contexts (McLin and Steward, 2006; Deacon et al., 2007; Mortazavi et al., 2021; Flynn et al., 2021). This includes variations between C57BL/6 and C57BL/10 strains in their resistance to seizure induction − likely to be related to functional differences in the brain glutamatergic system (McLin and Steward, 2006) − and a number of functional impairments, notably in spontaneous alternation tasks (Deacon et al., 2007). The two mouse strains also display significant variations across ∼2.800 distinct genes in single-nucleotide polymorphisms (SNPs) and copy number variations (CNVs) (Flynn et al., 2021; Mortazavi et al., 2022). Importantly, both SNPs and CNVs have been linked in genome-wide and other association studies to human neuropsychiatric/neurodevelopmental conditions and, more generally, to the expression of aberrant brain function phenotypes (McCarroll and Altshuler, 2007; Cook and Scherer, 2008; Merikangas et al., 2009; Morrow, 2010; Gratten et al., 2014; Brownstein et al., 2021; Rees and Kirov, 2021; Brownstein et al., 2022). More specifically, regarding the genetic background-dependent expression of muscular dystrophy phenotypes, it has been shown that skeletal muscle dysfunction due to loss of Dp427 may be more severe on the C57BL/6 genetic background (Beastrom et al., 2011). This may be considered as a putative confounding factor in behavioral tests with high motor demand, such as depression-related tests based on behavioral despair. A major contribution of motor factors to other tests performed in our present study is less likely. Dystrophic mouse lines may display reduced muscle force and increased muscle fatigue, altered respiratory and cardiac functions, reduced locomotion, and impairments in motor coordination (Goyenvalle et al., 2015; Aupy et al., 2020; Chesshyre et al., 2022). However, it has been shown that reduced locomotion during exploration of a new environment rather depends on stress reactivity (Vaillend and Chaussenot, 2017), and that freezing reflecting fear responses can be reduced by presentation of familiar olfactory cues (Yamamoto et al., 2010), thus pointing to altered behavioral processes rather than motor dysfunctions. Moreover, several studies have demonstrated that anxiety, fear responses and some cognitive deficits of dystrophic mice can be compensated by selective rescue of brain Dp427 (Sekiguchi et al., 2009; Zarrouki et al., 2022; Saoudi et al., 2023), which minimize potential contribution of peripheral factors.

In conclusion, our investigation sheds new light on the phenotypic expression of two different mutation profiles in DMD mouse models, and identifies robust outcome measures that were replicated between our laboratories. In light of their robustness against variability regarding housing and handling conditions, these measures should be considered as the most relevant neurobehavioral indexes to monitor in preclinical studies performed at different locations. We thus demonstrate that anxiety, fear-related behaviors and fear conditioning performance constitute a set of relevant outcome measures in the evaluation of novel therapies aimed at alleviating CNS comorbidities associated with loss of dystrophin isoforms in the brain. We also highlight the importance of using behavioral tests with low motor demands and standardized procedures, and the potential need to adapt test protocols according to different mouse genetic backgrounds. Overall, this work paves the way for further comparative studies of genotype−phenotype relationships in DMD, by providing key insights on the limits of reproducibility and interpretation of behavioral tests in DMD mouse models.

## MATERIALS AND METHODS

### Animals

Exon 52-deleted X-chromosome-linked muscular dystrophy model mice (*mdx52* mice) were produced by replacement of exon 52 of the *Dmd* gene with a neomycin resistance cassette, thereby eliminating expression of Dp427, Dp260 and Dp140 dystrophin isoforms but preserving expression of isoforms Dp116 and Dp71 (Araki et al., 1997). Breeders were generously provided by Dr Jun Tanihata and Dr Shin'ichi Takeda (National Center of Neurology and Psychiatry, Tokyo, Japan) to V.P.K. at Trinity College Dublin, Ireland (TCD) and C.V. at Paris-Saclay Institute of Neuroscience, France (NeuroPsi). The *mdx^5cv^* mice were originally generated by chemical mutagenesis (Chapman et al., 1989); they present a single A-to-T transversion in exon 10 of the dystrophin genomic DNA, creating a new splice donor site leading to a frameshifting 53 bp deletion and stop codon in the mRNA (Im et al., 1996). Breeders from the *mdx^5cv^* mouse line (B6Ros.Cg-*Dmd^mdx-5Cv^*/J) were purchased from the Jackson Laboratory (JAX stock #002,379; Bar Harbor, ME, USA) and transferred to Transpharmation Ireland (TIL) and NeuroPSI by TCD. The *mdx52* and *mdx^5cv^* mouse lines were backcrossed with the C57BL/6J parental strain for more than nine and 15 generations, respectively. For both lines, heterozygous females were crossed with C57BL/6JRj male mice (at NeuroPSI) or C57BL/6J male mice (at TCD), thus generating *mdx52* or *mdx^5cv^* and their littermate control males (wild-type, WT) to constitute experimental groups. Genotypes were determined by PCR analysis of tail DNA (at NeuroPSI) or ear biopsy (at TCD). Animal care and all experimental procedures complied with the European Communities Council Directive 86/609/EEC, EU Directive 2010/63/EU, the French National Committee decree 87/848, and the Irish Statutory Instrument 543/2012. Experimental protocols were additionally approved by the Paris-Sud and Centre Ethics Committee (CEEA N°59), the Irish Health Products Regulatory Authority (project authorization AE19136/P131), and the TCD Animal Research Ethics Committee.

### Experimental groups and general procedures

The behavior of *mdx52* and *mdx^5cv^* mice was investigated independently in two laboratories at TIL and NeuroPSI. The TIL and NeuroPSI acronyms are used in the next sections to discriminate protocols and results from the two laboratories. The batteries of tests performed in distinct cohorts of mice are represented in Fig. S1 and described below. Independent cohorts at NeuroPSI were submitted to single tests and are, therefore, not listed in this supplementary figure.

At NeuroPSI, male siblings were kept in groups (two to five animals per cage) under a 12-h light−dark cycle (light on 07:00) with food and water *ad libitum* and environmental enrichment (cardboard tunnel and sizzle nest). Individually ventilated cages (IVCs) were only used for breeding, while experimental mice were transferred to conventional cages after weaning. Behavioral testing was performed with observers being unaware of the genotype. A cohort of *mdx^5cv^* and WT littermates was tested for exploration and anxiety at the age of 2 months by being successively submitted to three behavioral tests, with intervals of 24 h between tests. Tests were carried out in the following order: (1) elevated plus maze, (2) light−dark choice, (3) open-field activity. The same cohort was submitted to the auditory-cued fear conditioning assay at 3 months of age. Distinct groups of 3–4-month-old *mdx^5cv^* and WT mice underwent the novel-object recognition test and depression-related tests. Distinct groups of *mdx52* mice and WT mice of the same age were used to test recognition memory in the novel object and spatial object recognition tests, and the delayed spontaneous alternation in a T-maze, as well as for assays investigating depression-associated phenotypes.

At TIL, male littermates were maintained in IVCs (Tecniplast, Italy) in groups of two to four animals per cage under controlled conditions (20−24°C, 45−65% relative humidity, 12:12 h light−dark cycle, light on 07:00, standard illumination 150 lux), with food and water available *ad libitum*. Cages included environmental enrichment, consisting of nesting material and one tunnel per cage. Behavioral testing was performed with observers being unaware of the genotype, and with a minimum interval between separate test paradigms of 24 h. For *mdx^5cv^* mice, a first cohort underwent the elevated zero maze, unconditioned fear response, and inverted screen grip tests at the ages of 6–7 weeks and 3–4 months. A second cohort was tested at 3–4 months of age in the T-maze delayed alternation assay at three different delay durations (1 h, 6 h, 24 h) and, subsequently, in the inverted screen grip test. A third cohort of 3–4-month-old mice was subjected first to the novel object recognition test using three delays (10 min, 30 min, 24 h), then to the T-maze delayed alternation assay (1 h, 6 h, 24 h delays) and, finally, to the inverted screen grip test. Concerning *mdx52* mice, one cohort was assessed at 6–7 weeks and 3–4 months of age in the elevated zero maze, light−dark choice, unconditioned fear response and inverted screen grip tests. Another cohort performed the novel object recognition test with three delays (10 min, 30 min, 24 h) and the inverted screen grip test at 3–4 months of age. A last cohort of 3–4-month-old mice underwent the open-field activity, forced swimming and inverted screen grip assays.

### Emotional reactivity

#### Open-field activity

The open field consisted in a square arena (50×50×50 cm) surrounded by black walls; the floor of the arena was covered with fresh cage-bedding material. Experiments were undertaken at constant room temperature (NeuroPSI: 22–23°C; TIL: 20–21°C) and homogeneous dim illumination (50 lux). Each mouse was released near the wall and video tracked for 25 min (at TIL) or 30 min (at NeuroPSI) by using ANY-maze (Stoelting, USA). Recorded XY positions were used to generate tracking plots of the exploration paths and to calculate parameters, such as distance travelled, average speed and time spent using either the entire apparatus or only using the central area of it (i.e. 10 cm from walls comprising a 30×30 cm area), referred to as the center of the open field. The percentage of time spent and distance traveled in the center zone were used as relative measures of anxiety.

#### Elevated plus maze

The maze consisted in two opposite-facing arms enclosed by high walls (closed arms, 20×8×25 cm), two opposite-facing open arms without walls (20×8 cm) and a central unenclosed area (8×8 cm) forming a plus sign, all situated above a vertical stand to elevate the maze 65 cm above the floor. Illumination was 150 lux in open and 30 lux in closed arms. At the start of a trial, mice were individually placed at the center of the maze with the head facing a closed arm. The number of entries and time spent in open or closed arms were then manually recorded during 5 min. Mice were considered to have entered one of the open sections of the maze if all four paws had crossed the threshold between closed and open areas. For automated scoring via ANY-maze, this criterion was defined as >90% of the body area of the animal being detected into the open section.

#### Elevated zero-maze

The apparatus consisted in an elevated ring-shaped runway with two opposite open (unwalled) and two opposite closed (walled) quadrants of equal dimensions (60 cm diameter, 5 cm corridor width, 16 cm wall height, 62 cm elevation from floor; Ugo Basile, Gemonio, Italy) illuminated by overhead lights at 500–600 lux (illumination in closed areas: 30–40 lux). A ceiling-mounted camera recorded the apparatus throughout testing for automated tracking and behavioral scoring using ANY-maze. At the start of a trial mice were placed in the center of a closed quadrant and were then allowed to freely explore the maze for 5 min. Animals were expected to avoid the aversive open arms, thus time spent, and entries made in the open arms were used as indexes of anxiety-like behavior. Mice were considered to have entered one of the open sections of the maze if all four paws had crossed the threshold between closed and open areas; for automated scoring via ANY-maze, this criterion was defined as >90% of an animal's body area being detected into the open section.

#### Light−dark choice

At NeuroPSI, the apparatus comprised a black Plexiglas-walled and dark compartment (15×15×20 cm; illumination: <15 lux) connected by a trap door (6×6 cm) to a brightly lit white Plexiglas-walled compartment (40×15×20 cm). Bright illumination was provided by a light source placed at the end of the lit compartment, opposite from the trap door, in order to create an illumination gradient (50 lux close to the trap door to 600 lux close to the light source). Each mouse was placed in the dark compartment for 10 s; the trap door was then opened and the mouse allowed to freely explore the whole apparatus for 5 min. Step through latency, number of entries and total time spent in the lit compartment were manually scored by the experimenter.

At TIL, the light−dark choice test was performed using Seamless Open-Field activity chambers (27×27×20 cm; Med Associates, Fairfax, VT, USA) equipped with three 16-beam infrared (IR) beam arrays located on both the X and Y axes for positional tracking and Z axis for rearing detection. The chambers were fitted with dark box inserts made of black IR-transparent acrylic, creating a dark enclosed compartment (13.5×27×20 cm; illumination: <10 lux) connected by a small door to an open compartment (13.5×27×20 cm) brightly lit by an overhead light source (illumination: 600 lux). Each mouse was placed at the center of the dark compartment from the hinged lid at the top of the insert. The mouse was then allowed to freely explore the entire apparatus for 5 min. Step through latency, number of entries and total time spent in the lit compartment, as well as other locomotor parameters, were automatically scored using the Activity Monitor 7 software (Med Associates). Setup schemes of the two paradigms used in our two laboratories are shown in Fig. S2B.

#### Unconditioned fear response

The mouse was restrained by grasping the scruff and back skin between thumb and index fingers, while securing the tail with the other fingers and tilting the animal upside-down so that the ventral part of its body faced the experimenter. After 15 s, the mouse was released to a novel transparent plastic cage (NeuroPSI: 24×19×12 cm, containing clean sawdust; TIL: 27×27×20 cm, containing no bedding) and was then video tracked for 5 min under homogeneous light conditions (NeuroPSI: 80 lux; TIL: 450 lux) using ANY-maze. Unconditioned fear responses induced by this short acute stress were characterized by periods of tonic immobility (freezing) and quantified during the 5-min recording period. Complete immobilization of the mouse, except for respiration, was regarded as a freezing response. The time spent freezing (in %) was calculated for group comparisons.

### Cognitive tests

#### Auditory-cued fear conditioning

The conditioning procedure and the StartFear (startle and fear) combined system (Panlab, Barcelona, Spain) were identical to those previously used in our studies of *mdx* and *mdx52* mice (Saoudi et al., 2021; Vaillend and Chaussenot, 2017). The conditioning chamber (25×25×25 cm) comprised three black methacrylate walls, a transparent front door and a grid floor connected to a shock scrambler to deliver footshocks as unconditioned stimuli (US), and a speaker mounted on the ceiling to deliver audible tones as conditioned stimuli (CS). The conditioning chamber rested on a high-sensitivity weight-transducer system to generate an analogical signal to reflect the movement of the animal. The chamber was confined to a ventilated soundproof enclosure (67×53×55 cm) on an anti-vibration table exposed to 60 dB white noise. Interchangeable floors and walls (i.e. plain floor and white walls) were used to analyze retention of cued fear in a novel context. To minimize stress before testing, *Mdx^5cv^* mice and WT littermates were gently handled every day for one week before being submitted to the task. On the first testing day (acquisition), a 2-min baseline period was recorded before delivery of five CS–US pairings, i.e. five CS (80 dB at 10 kHz for 30 s per stimulus) paired with five US (0.4 mA for 2 s per stimulus), with variable and pseudo-randomly distributed intervals (60, 120 or 180 s) between each CS–US pair. On the second day (retention), the session started by placing the mouse in a different context for 2 min (baseline) before delivering four CS (80 dB at 10 kHz, 30 s) separated by intervals of variable durations (60, 90 or 120 s). Movements of all animals tested were sampled at 50 Hz for quantitative analysis (FREEZING software, Panlab). Freezing was analyzed during delivery of the CS (30 s) to specifically reflect associative learning performance (Chaussenot et al., 2015).

#### Delayed spatial alternation in a T-maze

At NeuroPSI, the apparatus was made of transparent Plexiglas walls, composed of a central alley (40×10×25 cm) including a start box (15×10×25 cm) and two lateral alleys (30×10×25 cm) positioned at the end of the central alley. Sliding transparent doors were placed at the entrance to each alley (central or lateral). Illumination inside the apparatus was 50 lux. At TIL, the T-maze (Multimaze, Ugo Basile), made in a gray non-reflective material, consisted of a central alley (35×5×12 cm) connected to a start box (15×5×12 cm) on one end, and a square arena attached to two side arms (35×5×12 cm) positioned at a 90° angle from the central corridor at its other end. The entire maze was raised 42 cm from the floor, and sliding doors controlled remotely via ANY-maze were located between the start box and the central alley, and at the entrance of each corridor at central arena level.

On the first day of testing, each animal was allowed to explore the maze for 5 min in a single habituation trial. On the following day, mice were submitted to two consecutive acquisition trials during which they had access to only one lateral alley, the other being closed by a sliding door; half of the animals for each experimental group had access to the right arm, and the other half to the left arm of the T-maze. At the start of each trial, the animal was placed for 30 s in the start box, and all sliding doors except for the blocked side arm were then opened. The mouse was allowed 10 min to explore the maze; once it had entered the accessible lateral alley (with an entry being determined by all paws crossing the door's threshold), it was confined in this alley for 30 s by closure of the correspondent lateral sliding door; subsequently, the mouse was submitted to a second trial under the same conditions. At TIL, in case of no movement/no entry into the open side arm within 2 min from trial start, the acquisition trial was restarted after providing the animal a brief resting interval. After 1, 6 or 24 h, each animal underwent a retention trial during which, after opening of the start box door, it could choose freely between the left and the right alley. During retention testing at TIL, maximum duration for each trial was 2 min; no movement or no entry in either choice arm within this timeframe was counted as a failed trial. Mice that underwent testing for multiple delays repeated the acquisition and retention protocols with a minimum interval of 5 days between testing sessions, to minimize the influence of previously acquired spatial memories. The position of the acquisition arm was alternated between successive testing with different delays. For each experimental group, retention of spatial memories from the two acquisition trials was expressed by the percentage of mice alternating (i.e. exploring the previously inaccessible arm) during the final trial. The latencies to enter the lateral alley during the first two trials (acquisition trials) and during the third trial (retention testing) were additionally recorded and compared between genotypes.

#### Spatial and non-spatial object recognition

At NeuroPSI, the test arena consisted of a square open field (50×50×50 cm) with black walls and a white floor covered with sawdust. At TIL, the apparatus was a square open-field arena (40×40×40 cm) with gray walls and a floor covered with cage-bedding material. Experiments were undertaken at constant room temperature (NeuroPSI: 22–23°C; TIL: 20–21°C) and homogeneous dim illumination (40–50 lux). Several procedures were used to address object recognition under different conditions, including the procedure originally used to characterize the recognition memory in the Dp427-deficient *mdx* mouse (Vaillend et al., 2004). In all experiments, mutant model mice and WT littermates were gently handled every day for a week to minimize stress before testing. Experimenters from the two laboratories were unaware of the genotype, and clear criteria for recording and analyzing behavioral responses were established at the outset of this study. Moreover, data from all experimenters (two per laboratory) were compared within and between laboratories to maximize accuracy and reliability of the analyses.

##### Procedure 1 − two different objects during acquisition

The testing procedure (Vaillend et al., 2004) started with a 4-day habituation period consisting of two daily 10-min sessions separated by a 5-h delay. On the first day, littermates from a given cage were placed simultaneously in the empty apparatus and allowed to freely explore the arena for 10 min. On the subsequent days, mice were exposed individually to the empty open field to let them familiarize with the apparatus and to record spontaneous locomotor activity. On the last pretraining session (day 4), two identical basic plastic objects were placed in the box and mice were allowed to freely explore the objects for 10 min. These two objects were not used for subsequent memory testing. The object recognition test started 48 h after the end of the habituation period. At NeuroPSI, mice were first submitted to a single acquisition trial during which they were exposed for 10 min to two different objects, placed on the midline of the open field at 15 cm from the walls (objects spaced by ∼15−18 cm). At TIL, mice underwent an acquisition session comprising three 5 min trials, with an intertrial interval of 2−3 min, during which mice could freely investigate two discrete objects placed on the midline of the open field at 10 cm from the walls (objects spaced by ∼15 cm). Memory retention was tested after delays of 10 min or 24 h; in addition, TIL performed memory testing after a 30 min delay. During the retention session, a novel object replaced one of the objects used during acquisition, and mice were allowed to explore the arena for 5 min. The next day, mice were submitted to a second acquisition phase with another set of objects and recognition memory was tested at a different retention delay. This procedure was repeated to assess a third delay duration at TIL. Each subject was thus submitted to two or three successive acquisition/retention phases, following a sequence of retention delays that was counterbalanced among individuals. In case memory was assessed at an additional delay of 30 min, testing with this acquisition/retention interval was performed at the end of testing for the 10 min or 24 h delays.

##### Procedure 2 − two identical objects during acquisition

After a recovery period of 72 h, the same mice tested at NeuroPSI underwent a second 4-day habituation period and were then submitted to the same testing protocol (two acquisition sessions, each followed by a retention session at 10 min or 24 h delay), except that the sets of two objects used for the acquisition session were identical.

##### Procedure 3 − non-spatial and spatial object recognition with three different objects during acquisition

The testing procedure was performed at NeuroPSI. It started with a shorter period of habituation, to reduce the climbing behavior observed in other experiments with longer periods of habituation. Habituation, thus, consisted of a single day with a 10-min session, during which mice from a given cage were placed simultaneously in the empty apparatus. This was followed 4 h later by a 10-min exploration session, during which mice were placed individually in the open field. The habituation was followed by a day off before the start of the testing procedure. Mice were then submitted to an acquisition session consisting of three successive 5-min trials with 5 min intertrial intervals, during which they were exposed to three different objects, placed at 15 cm from two adjacent walls and forming an isosceles right triangle with two 15−18 cm-long sides. Spatial memory retention was tested 24 h later, by moving one of the objects used during acquisition towards the opposite side of the apparatus (spatial object recognition). Mice were allowed to explore for 5 min. After a day off, animals were submitted to another acquisition session of three successive trials with objects at the same position as for the previous retention session. During the retention session 24 h later, a novel object was used to replace one of those of the acquisition session [novel object recognition (NOR)]. Each mouse was, thus, tested twice, i.e. under the spatial and non-spatial condition, following a sequence that was counterbalanced among individuals.

##### Procedure 4 − two different objects with shorter habituation and longer acquisition

This procedure was only used to test *mdx^5cv^* mice at NeuroPSI, in an attempt to reduce the object and wall climbing behavior observed in other experiments. Here, mice were submitted to a shorter period of habituation (1 day followed by a day off), as for the spatial/non-spatial test above, and then to an acquisition session consisting of three trials of 5 min with 5-min intertrial intervals, during which they were exposed to two different objects. Memory retention was tested 24 h later by replacing one of the objects used during acquisition by a novel object.

##### Data acquisition and analysis

A video of each trial per animal was recorded using ANY-maze. During the acquisition and retention phases, the time spent by the mouse in contact with the objects was manually scored by the experimenter using event recorder keys. A contact was defined as the paws, snout or vibrissae of the animal touching the object, excluding any climbing on top of the object to assess the surrounding environment. Mice that had explored each object <4 s during acquisition phases were considered being outliers and were not tested further (NeuroPSI) or excluded from analysis (TIL) in the retention phase. A recognition index was calculated for each subject based on object exploration times recorded in the retention trial (novel object exploration time ×100/total object exploration time) and object recognition was evaluated by comparing the relative exploration of the novel object to chance level (50%) or by comparing exploration measures of the familiar and novel objects.

### Depressive-like behaviors

#### Tail suspension test

Each mouse was suspended 35 cm above a bench top for 6 min by placing adhesive tape 2 cm from the tip of the tail (Vaillend and Chaussenot, 2017). Behavior was recorded on video during two sessions separated by a 24 h delay. The latency to the first bout of immobility (freezing latency) and the duration of freezing were quantified offline using event-recorder keys in ANY-maze. Complete immobility for >2 s was regarded as freezing.

#### Forced swimming test

Each mouse was lowered in an inescapable glass cylinder [diameter: 11 cm (NeuroPSI) or 13.5 cm (TIL); height: 23 cm (NeuroPSI) or 28 cm (TIL)] filled with water (25±1°C) to 18 cm from the bottom of the cylinder (Vaillend and Chaussenot, 2017). Room temperature was 25°C (NeuroPSI) or 21°C (TIL). Behavior in the cylinder was recorded on video for 5 min in two sessions separated by a 24 h delay. Video files were analyzed offline using event recorder keys in the behavior tracking and scoring software ANY-maze to quantify the latency and duration of the three main parameters climbing, staying afloat and immobility (freezing). Climbing was considered when mice had a vertical position of the spine with the forepaws striking the glass walls while hind paws showed repetitive movement in water. Staying afloat corresponded to small movements simply performed to keep the head above water. Immobility was defined by a complete immobilization of the body for at least 1 s. The time not spent performing any one of these activities represented either unspecific uncoordinated movements or swimming activity involving horizontal spine position with legs treading water and producing a clear displacement of the body.

### Statistics

All statistical analyses were performed using GraphPad Prism 9 (GraphPad Software). Mann–Whitney *U* test was employed to compare individual behavioral outcome variables between two genotype groups. Each mouse line was always compared to its respective littermate control groups. When two lines were tested following the same protocol, using the same facility and by the same experimenter, comparisons of the two mutant lines and WT line were performed using Kruskal–Wallis one-way ANOVA on ranks followed by Dunn's test. In assays comprising multiple trials across one testing session or across time-points (e.g. novel object recognition test, cued fear conditioning), data were compared by using two-way ANOVA (factors: genotype, trial) with repeated measures on one factor (trial), or with residual maximum likelihood (REML) mixed-effects model in case of missing values, followed by Holm−Šídák post hoc tests for multiple comparisons. T-maze alternation rates, expressed as the proportions of mice alternating (in %), were compared using Chi-squared analysis. Wilcoxon signed-rank tests were used to compare recognition indexes against chance levels in object recognition tests.

## Supplementary Material

10.1242/dmm.050707_sup1 Supplementary information
