# Supplementary material for: Impact of distinct dystrophin gene mutations on behavioral phenotypes of Duchenne muscular dystrophy
Source: Dis Model Mech. 2024 Dec 24;17(12):dmm050707. doi: 10.1242/dmm.050707 (PMC11698058; doi:10.1242/dmm.050707)
Supplement: Supplementary information [file dmm-17-050707-s1.pdf]

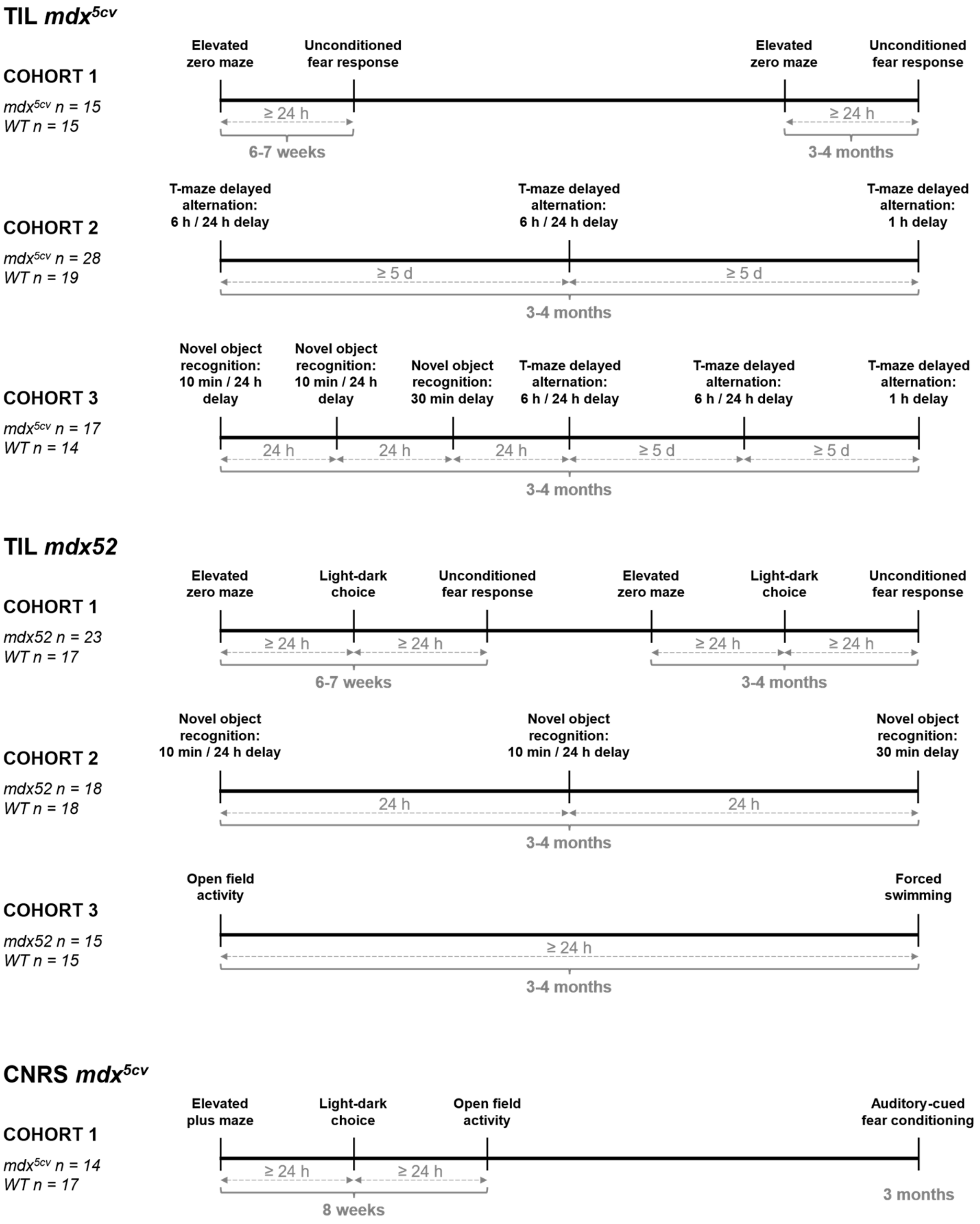

Fig. S1. Schematic representation of the experimental design for mouse cohorts. Diagrams show animals' ages and illustrate the sequences of tests and intervals between tests performed at TIL (mdx5cv: 3 cohorts; mdx52: 3 cohorts) and NeuroPSI (last cohort of mdx5cv). All other tests were performed using independent cohorts of naïve mice and are not shown here.

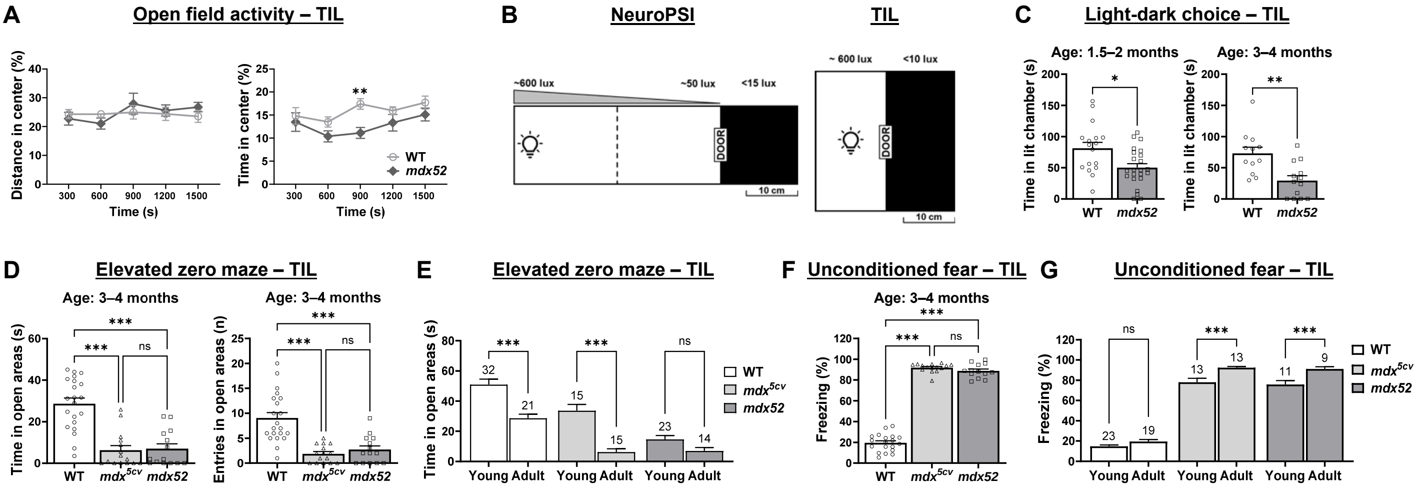

Fig. S2. Effects of age, retesting and environment on emotional responses in *mdx^{5cv}* and *mdx52* mice. (A) Open field activity assay in 3–4 months old *mdx52* mice (n=15) and their wild-type (WT) littermates (n=15) tested at TIL. Emotional reactivity (anxiety-like behaviour) was estimated by the percentage of time spent and percentage of distance travelled in the centre zone of the arena. (B) Schematic representation of the light-dark choice test apparatus (top views) at NeuroPSI and TIL labs. At NeuroPSI the testing arena was composed of a dark box (15×15 cm), in which each mouse was first introduced, and a lit box (40×15 cm). The two compartments were connected by a sliding door. The lit box was separated in half by a virtual line (dotted line) to analyze behavior in door-adjacent and door-distal parts of this box, which had different light intensities; the light intensity gradient is shown above the drawing. The door-distal part of the lit box (600 lx) is considered more anxiogenic than the door-adjacent area (50 lx). At TIL the dark (10 lx) and lit boxes (600 lx) were of equivalent size and were separated by an open door. (C) At TIL, a group of *mdx52* (n=23) and WT (n=17) mice underwent the light-dark choice at 1.5–2 months of age; part of these *mdx52* (n=13) and WT (n=12) animals were further retested at 3–4 months of age. Plots show behavioral anxiety levels expressed by the time spent in the lit chamber of the test arena. (D) A portion of the animals previously tested in the elevated zero maze at 1.5–2 months of age (see Fig. 1D) were retested at 3–4 months of age: *mdx^{5cv}* (n=15), *mdx52* (n=14) and WT (n=21) mice. Plots show the time spent and number of entries made in open areas compared between genotype groups. (E) Comparison of time spent in open areas of the elevated zero maze in WT, *mdx^{5cv}*, a n d *mdx52* mice first tested at 1.5–2 months of age ("Young"; see Fig. 1D) and those retested once at 3–4 months old ("Adult"). (F) A portion of the animals previously tested for the unconditioned fear response (see Fig. 1E) were retested at 3–4 months of age: *mdx^{5cv}* (n=13), *mdx52* (n=9) and WT (n=19) mice. Histograms show the percentage of time spent in tonic immobility (% freezing) during a 5-min period following a brief scruff restraint (15 s) across genotype groups of 3–4 months old mice. (G) Comparison of the percentage of freezing in mice first tested at 1.5–2 months of age ("Young"; see Fig. 1E) and in those retested once at 3–4 months old ("Adult"). Data are presented as mean + SEM. In D and E the performance of the WT animals from the two cohorts were pooled following verification that their performance was statistically comparable. Statistical analyses: two-way RM ANOVA followed by Šídák's multiple comparisons tests (A), Mann-Whitney U test (C), Kruskal–Wallis followed by Dunn's multiple comparisons tests (D & F), and two-way ANOVA followed by Fisher's LSD pairwise comparison test (E & G). *P<0.05; **P<0.01; ***P<0.001; ns, not significant.

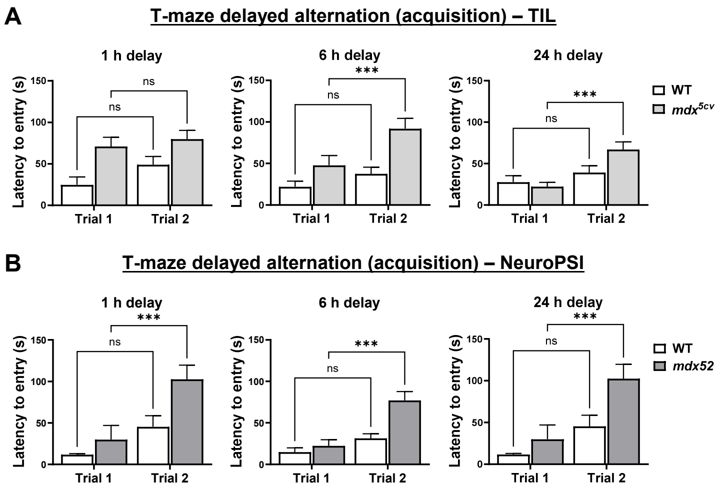

Fig. S3. Latencies to lateral arm entry during acquisition trials in the T-maze delayed alternation test. (A) 3–4 months old *mdx*^{5cv} mice (n=39) and their wild-type (WT) littermates (n=31) tested at TIL. (B) 3–4 months old *mdx52* mice (n=22) and their WT littermates (n=18) tested at NeuroPSI. Data are presented as mean + SEM. Statistical analyses: two-way RM ANOVA followed by Šídák's multiple comparisons test; ***P<0.001; ns, not significant.

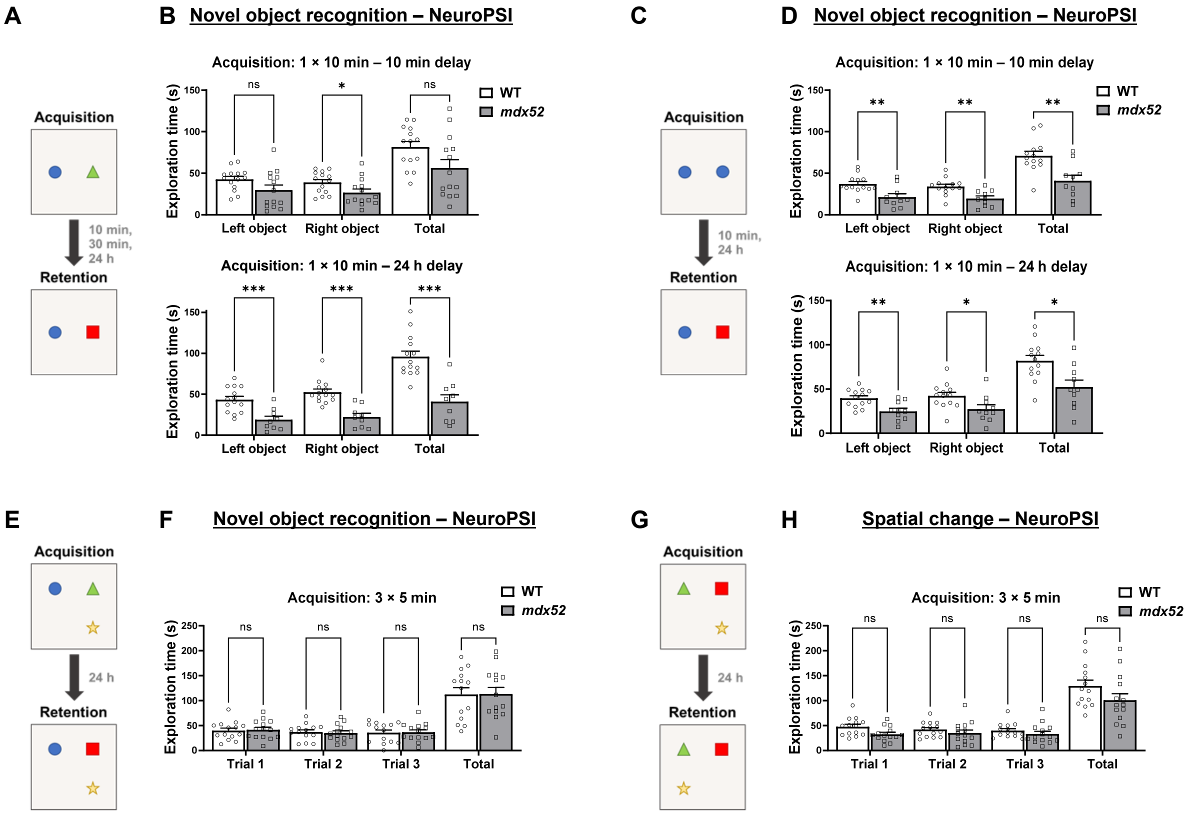

Fig. S4. Exploration time of *mdx52* mice during acquisition sessions of novel object recognition tests and recognition of object's spatial change test. All histograms show time spent exploring objects during the acquisition sessions of novel object and spatial object recognition tests performed at NeuroPSI with 3–4 months old *mdx52* mice (n=16) and their WT littermates (n=14)(memory performance described in Fig. 3). Schematic representations of the different protocols are shown for protocol with two different objects (A), two identical objects (C) sets at acquisition, three different objects sets at acquisition (E), and for the spatial object change with three different objects set at acquisition (G). The corresponding object exploration time graphs are shown on the right of the schematic representations. Exploration time of the left, right and both objects (total) are shown for the tests using two different objects set configurations (B, D). Exploration time of the sum of three objects are shown during the three successive acquisition trials and all trials (total) for the tests using three different objects set configurations (F, H). Data are presented as mean + SEM. Statistical analyses: Mann-Whitney U test (B, D, F, H), two-way ANOVA followed by Šídák's multiple comparisons test across multiple acquisition trials (F & H). *P<0.05; **P<0.01; ***P<0.001; ns, not significant.

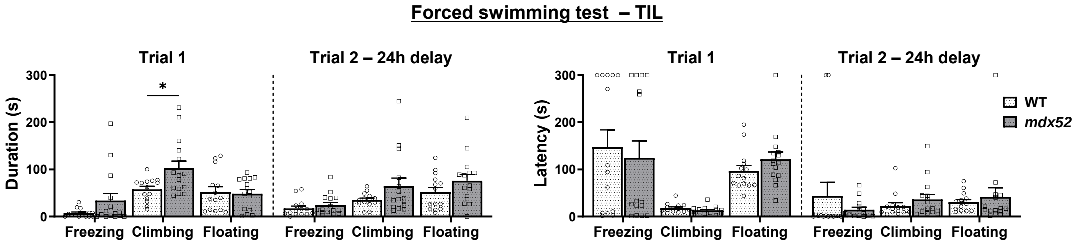

Fig. S5. Behavioral despair and learned helplessness in *mdx52* mice during forced swimming test at TIL. Freezing, climbing and floating durations, as well as the latencies at which these behaviors were first observed, were measured during two successive trials 24 h apart in 3–4 months old *mdx52* (n=15) and WT littermates (n=15). Data are presented as mean + SEM. Statistical analyses: two-way RM ANOVA followed by Šídák's multiple comparisons tests. *P<0.05; all other comparisons were not significant.

Table S1. Details of statistical analyses presented in main and supplementary figures. All statistics indicate the main genotype effect. Significant statistics are highlighted in bold font.

Figure	Test	Parameter	Statistical test	P value	F / U / H
**Main figures**					
Figure 1	Open field activity	NeuroPSI - % Distance mdx^{5cv}	Two-way RM ANOVA	0.1390	F (1, 29) = 2.315
		NeuroPSI - % Time mdx^{5cv}	Two-way RM ANOVA	0.8956	F (1, 29) = 0.018
	Light-dark choice	NeuroPSI - Time mdx^{5cv}	Mann-Whitney	**0.0357**	U = 37
		NeuroPSI - Entries mdx^{5cv}	Mann-Whitney	**0.0481**	U = 39.5
	Elevated plus maze	% Time mdx^{5cv}	Mann-Whitney	**0.0010**	U = 15
		% Entries mdx^{5cv}	Mann-Whitney	**0.0202**	U = 29
	Elevated zero maze	Time mdx^{5cv}, *mdx52* (Young)	Kruskal-Wallis	**<0.0001**	H (2) = 33.18
		Entries mdx^{5cv}, *mdx52* (Young)	Kruskal-Wallis	**<0.0001**	H (2) = 26.97
	Unconditioned fear response	% Freezing mdx^{5cv}, *mdx52* (Young)	Kruskal-Wallis	**<0.0001**	H (2) = 34.64
	Cued fear conditioning	Acquisition: % Freezing mdx^{5cv}	Two-way RM ANOVA	**0.0075**	F (1, 29) = 8.261
		Retention: % Freezing mdx^{5cv}	Two-way RM ANOVA	**0.0002**	F (1, 29) = 18.69
Figure 2	T-maze delayed alternation	TIL - Arm choice latency mdx^{5cv}	Two-way RM ANOVA	**0.0010**	F (1, 69) = 11.44
		NeuroPSI - Arm choice latency *mdx52*	Two-way RM ANOVA	**0.0052**	F (1, 114) = 8.133
		Arm choice latency mdx5cv vs mdx52	Two-way RM ANOVA	**0.0321**	F (1, 60) = 4.817
Figure 3	Novel object recognition (two different objects)	TIL - RI *mdx52*	Two-way RM ANOVA	0.1999	F (1, 34) = 1.709
		TIL - RI mdx^{5cv}	Two-way RM ANOVA	0.6962	F (1, 29) = 0.1555
		TIL - RI mdx^{5cv} vs *mdx52*	Two-way RM ANOVA	0.5784	F (1, 33) = 0.3150
		NeuroPSI - RI *mdx52*	Two-way RM ANOVA	0.6238	F (1, 52) = 0.2434
		NeuroPSI - RI mdx^{5cv}	Mann-Whitney	0.4323	U = 91
		NeuroPSI - RI mdx^{5cv} vs *mdx52*	Mann-Whitney	0.3622	U = 66
	Novel object recognition (two similar objects)	RI *mdx52*	Two-way RM ANOVA	0.9687	F (1, 28) = 0.001567
	Novel object recognition (three different objects)	RI *mdx52*	Mann-Whitney	0.1371	U = 65
	Spatial object recognition (three different objects)	RI *mdx52*	Mann-Whitney	0.9459	U = 96
Figure 4	Tail suspension test	Freezing time mdx^{5cv}	Two-way RM ANOVA	0.6789	F (1, 28) = 0.1750
		Freezing latency mdx^{5cv}	Two-way RM ANOVA	**0.0352**	F (1, 28) = 4.896
		Freezing time *mdx52*	Two-way RM ANOVA	0.8914	F (1, 38) = 0.01889
		Freezing latency *mdx52*	Two-way RM ANOVA	**0.0072**	F (1, 38) = 8.074
		Freezing time mdx^{5cv} vs *mdx52*	Two-way RM ANOVA	0.7094	F (1, 34) = 0.1412
		Freezing latency mdx^{5cv} vs *mdx52*	Two-way RM ANOVA	0.5608	F (1, 34) = 0.3451
	Forced swimming test	NeuroPSI - Freezing duration mdx^{5cv}	Two-way RM ANOVA	**0.0062**	F (1, 28) = 8.770
		NeuroPSI - Climbing duration mdx^{5cv}	Two-way RM ANOVA	0.5830	F (1, 28) = 0.3085
		NeuroPSI - Floating duration mdx^{5cv}	Two-way RM ANOVA	**0.0015**	F (1, 28) = 12.43
		NeuroPSI - Freezing latency mdx^{5cv}	Two-way RM ANOVA	**<0.0001**	F (1, 28) = 79.48
		NeuroPSI - Climbing latency mdx^{5cv}	Two-way RM ANOVA	0.4072	F (1, 28) = 0.7081
		NeuroPSI - Floating latency mdx^{5cv}	Two-way RM ANOVA	**0.0010**	F (1, 28) = 13.60
		NeuroPSI - Freezing duration *mdx52*	Two-way RM ANOVA	**0.0050**	F (1, 32) = 9.092
		NeuroPSI - Climbing duration *mdx52*	Two-way RM ANOVA	0.9108	F (1, 32) = 0.01274
		NeuroPSI - Floating duration *mdx52*	Two-way RM ANOVA	0.7012	F (1, 32) = 0.1499
		NeuroPSI - Freezing latency *mdx52*	Two-way RM ANOVA	**<0.0001**	F (1, 32) = 25.30
		NeuroPSI - Climbing latency *mdx52*	Two-way RM ANOVA	0.4672	F (1, 32) = 0.5414
		NeuroPSI - Floating latency *mdx52*	Two-way RM ANOVA	0.1595	F (1, 32) = 2.075
		NeuroPSI - Freezing duration mdx^{5cv} vs *mdx52*	Two-way RM ANOVA	0.3048	F (1, 26) = 1.096
		NeuroPSI - Climbing duration mdx^{5cv} vs *mdx52*	Two-way RM ANOVA	0.4454	F (1, 26) = 0.6005
		NeuroPSI - Floating duration mdx^{5cv} vs *mdx52*	Two-way RM ANOVA	**0.0448**	F (1, 26) = 4.447
		NeuroPSI - Freezing latency mdx^{5cv} vs *mdx52*	Two-way RM ANOVA	0.0652	F (1, 26) = 3.708
		NeuroPSI - Climbing latency mdx^{5cv} vs *mdx52*	Two-way RM ANOVA	0.8841	F (1, 26) = 0.02167
		NeuroPSI - Floating latency mdx^{5cv} vs *mdx52*	Two-way RM ANOVA	0.3123	F (1, 26) = 1.062
**Supplementary figures**					
Figure S2	Open field activity	TIL - % Distance *mdx52*	Two-way RM ANOVA	0.8092	F (1, 28) = 0.05940
		TIL - % Time *mdx52*	Two-way RM ANOVA	**0.0118**	F (1, 28) = 7.257
	Light-dark choice	TIL - Time *mdx52* (Young)	Mann-Whitney	**0.0105**	U = 103
		TIL - Time *mdx52* (Adult)	Mann-Whitney	**0.0052**	U = 28
	Elevated zero maze	Time mdx^{5cv}, *mdx52* (Adult)	Kruskal-Wallis	**<0.0001**	H (2) = 25.83
		Entries mdx^{5cv}, *mdx52* (Adult)	Kruskal-Wallis	**<0.0001**	H (2) = 26.26
		Time mdx^{5cv}, *mdx52* Young vs Adult	Two-way ANOVA	**<0.0001**	F (2, 114) = 42.05
	Unconditioned fear response	% Freezing mdx^{5cv}, *mdx52* (Adult)	Kruskal-Wallis	**<0.0001**	H (2) = 34.01
		% Freezing mdx^{5cv}, *mdx52* Young vs Adult	Two-way ANOVA	**<0.0001**	F (2, 82) = 625.2
Figure S3	T-maze delayed alternation	TIL - 1 h Arm choice latency mdx^{5cv}	Two-way RM ANOVA	**0.0008**	F (1, 68) = 12.33
		TIL - 6 h Arm choice latency mdx^{5cv}	Two-way RM ANOVA	**0.0019**	F (1, 65) = 10.52
		TIL - 24 h Arm choice latency mdx^{5cv}	Two-way RM ANOVA	0.2049	F (1, 68) = 1.638
		NeuroPSI - 1 h Arm choice latency *mdx52*	Two-way RM ANOVA	**0.0451**	F (1, 38) = 4.293
		NeuroPSI - 6 h Arm choice latency *mdx52*	Two-way RM ANOVA	**0.0058**	F (1, 38) = 8.554
		NeuroPSI - 24 h Arm choice latency *mdx52*	Two-way RM ANOVA	**0.0451**	F (1, 38) = 4.293
Figure S4	Novel object recognition (two different objects)	Exploration time left - 10 min delay *mdx52*	Mann-Whitney	0.0916	U = 61
		Exploration time right - 10 min delay *mdx52*	Mann-Whitney	**0.0205**	U = 48
		Exploration time total - 10 min delay *mdx52*	Mann-Whitney	0.0511	U = 55.5
		Exploration time left - 24 h delay *mdx52*	Mann-Whitney	**0.0009**	U = 13
		Exploration time right - 24 h delay *mdx52*	Mann-Whitney	**<0.0001**	U = 5
		Exploration time total - 24 h delay *mdx52*	Mann-Whitney	**0.0002**	U = 8
	Novel object recognition (two similar objects)	Exploration time left - 10 min delay *mdx52*	Mann-Whitney	**0.0065**	U = 22
		Exploration time right - 10 min delay *mdx52*	Mann-Whitney	**0.0025**	U = 18
		Exploration time total - 10 min delay *mdx52*	Mann-Whitney	**0.0065**	U = 22
		Exploration time left - 24 h delay *mdx52*	Mann-Whitney	**0.0043**	U = 20.5
		Exploration time right - 24 h delay *mdx52*	Mann-Whitney	**0.0214**	U = 28
		Exploration time total - 24 h delay *mdx52*	Mann-Whitney	**0.0147**	U = 26
	Novel object recognition (three different objects)	Exploration time by trial- 24 h delay *mdx52*	Two-way RM ANOVA	0.9741	F (1, 26) = 0.001077
		Total exploration time - 24 h delay *mdx52*	Mann-Whitney	0.7688	U = 91
	Spatial object recognition (three different objects)	Exploration time by trial - 24 h delay *mdx52*	Two-way RM ANOVA	0.1155	F (1, 26) = 2.652
		Total exploration time - 24 h delay *mdx52*	Mann-Whitney	0.0849	U = 60
Figure S5	Forced swimming test	TIL - Freezing duration *mdx52*	Two-way RM ANOVA	0.0906	F (1, 27) = 3.081
		TIL - Climbing duration *mdx52*	Two-way RM ANOVA	**0.0315**	F (1, 27) = 5.148
		TIL - Floating duration *mdx52*	Two-way RM ANOVA	0.3770	F (1, 27) = 0.8068
		TIL - Freezing latency *mdx52*	Two-way RM ANOVA	0.4391	F (1, 27) = 0.6167
		TIL - Climbing latency *mdx52*	Two-way RM ANOVA	0.4997	F (1, 27) = 0.4681
		TIL - Floating latency *mdx52*	Two-way RM ANOVA	0.3210	F (1, 27) = 1.022
